# Does the range of ecolabels on offer alter their impact on meal selection? An online randomised control trial

**DOI:** 10.1186/s12889-025-25870-8

**Published:** 2025-12-13

**Authors:** Elizabeth Biggs, Emma E. Garnett, Rachel Pechey

**Affiliations:** Nuffield Department of Primary Care Health Sciences, University of Oxford, Radcliffe Observatory Quarter, Oxford, OX2 6GG UK

**Keywords:** Ecolabels, Moderators, Behaviour change, Environmentally sustainable food

## Abstract

**Background:**

Environmentally sustainable meals might be more likely to be selected when ecolabels indicating environmental impact are present, but the effectiveness of ecolabels may depend on the range of meal options available (e.g. their comparative environmental impact).

**Methods:**

We ran a proof-of-principle study to test whether changing the range of ecolabel values (A-E: lowest – highest impact) shown in hypothetical meal selection scenarios affected sustainable meal choices. In a randomised controlled trial, all participants were presented with five themed meal selection scenarios, which contained three realistic meal options (e.g. a vegetarian, chicken, or beef burger) from which they selected their preferred option. We randomised a sample of the UK population (*n* = 2646) to a control (no ecolabels present) or intervention (ecolabels present) group. Participants in the intervention group were randomised to see different combinations of ecolabels assigned to each meal, in each scenario. For example, one participant could see ecolabels ‘A’, ‘C’ and ‘E’ assigned to meals in one scenario, whilst another may see ‘B’, ‘C’, ‘D’.

**Results:**

We found that whilst ecolabels discouraged selection of the least sustainable meals in comparison to control (no ecolabels present), changing the range of ecolabels on offer had little effect on sustainable meal selection. The only exception was that participants who saw ‘C’ ecolabels uniformly assigned to each meal had significantly lower odds of choosing a sustainable item than participants who saw the widest range of ecolabels (‘A’, ‘C’ and ‘E’) (OR for selecting the lowest impact product (vs. medium or highest impact products): 0.71, CI: 0.58–0.88, *p* = 0.001).

**Conclusions:**

We found that the widest range of ecolabels is more effective than the narrowest, with limited evidence on the impact of intermediate ranges.

**Trial registration:**

Registered 6/6/2023 on the Open Science Framework (https://osf.io/69xct/).

**Supplementary Information:**

The online version contains supplementary material available at 10.1186/s12889-025-25870-8.

## Introduction

Transformations towards environmentally sustainable diets are urgently required to stabilise the biosphere and prevent further biodiversity loss [1]. To achieve sustainable and healthy diets in high income countries, food environments are needed that support consumers to reduce their consumption of food types with the highest environmental impacts, such as animal derived foods [1, 2]. One potential policy, which has relatively high public support [3, 4], comprises placing ecolabels on food products to communicate information about the relative environmental sustainability of different food products. In doing so, ecolabels provide knowledge to consumers, correcting information asymmetries between producers and consumers, and potentially changing consumer behaviour [5]. Providing information to consumers to promote environmentally sustainable consumption patterns is highlighted by Target 15 of the Global Biodiversity Framework [6] and therefore governments that have agreed to the Global Biodiversity Framework may encourage businesses to implement ecolabels through legal, administrative or policy measures. However, ecolabels must be effectively implemented if they are to contribute to sustainable dietary shifts.

In the food sector, labelling is well-established for purposes of communicating nutritional information, quality assurance, and food safety [7]. Within Europe, the use of environmental sustainability claims and ecolabels on newly introduced products is increasing [5]. However, sustainability labelling has been typically limited to certification schemes with a particular social or ecological focus (e.g. Fairtrade, Marine Stewardship Council), or single aspect of environmental sustainability (e.g. greenhouse gas emissions or pesticide use). This is evidenced by a systematic review assessing 76 ecolabelling interventions across 56 studies, of which many focussed on a single aspect of environmental sustainability [8]. However, the review provided preliminary evidence that ecolabels could promote the selection, purchase and consumption of more sustainable food and drinks [8]. Since this review, online studies looking at composite labels (i.e. a single summary environmental impact score) have suggested these may be effective at encouraging more sustainable food selections [9, 10] when placed on individual food products, but not in the form of badges indicating environmental impact across the shopping basket [11]. Given the 2021 French legislation regarding environmental labelling of food, which states that not only climate change, but other impacts such as water consumption should be considered [12], further research into composite environment labels is needed.

In the UK, ecolabels that use traffic light labelling are currently favoured by the Institute of Grocery Distribution for recommended implementation in the UK [13], and can be used to communicate composite environmental scores. Traffic light labelling is characterised by a colour-coded system (from red – green) and are commonly used to communicate the nutritional content of food. For example, in France, food products are commonly labelled with “Nutri-Scores”, which use a five-level ordinal scale with traffic light colours to denote the overall nutritional value of a food product [12] and have been shown to be effective and equitable [14]. Like nutritional scores, traffic light labelling can be used to communicate the overall environmental impact of food. Traffic-light ecolabels have been evaluated in real world purchasing environments, with evidence suggesting that red-yellow-green labels reduce the proportion of meat dishes purchased in university cafeterias [15], but this effect was not found in a randomised controlled trial of traffic light ‘A’ – ‘E’ (red-green) labels across worksite cafeterias, catering to a wider demographic [3]. In addition, compared to many of the online studies, the field trial menus offer a limited range of ecolabels on a given day, and it is possible a small change (e.g. an ecolabel value difference from ‘A’ to ‘B’ or ‘D’ to ‘E’) may not offer sufficient perceived benefit in terms of environmental impact to overcome a habitual meal selection. When a larger range of ecolabelled items is available, there is greater potential for increasing or reducing the environmental impact of meal purchases. For example, a range of meal options involving meals labelled ‘A’, ‘C’, and ‘E’ could lead to a switch from ‘E’ to ‘A’, whereas for a smaller range of ‘C’, ‘C’, and ‘D’, the maximum switch is from ‘D’ to ‘C’. In the worksite cafeteria study around half of all food items sold were labelled ‘E’, with evidence from fidelity check photographs showing some instances where all the hot main meal options available were rated ‘E’ [3]. This leaves open the possibility that if a larger range of ecolabel scores on meal options had been available, there may have been increased selection of low impact meal options.

Increasing the range of label types on offer may increase sustainable meal purchases. For example, labelling products with red, orange, and green labels has been demonstrated to be more effective at increasing sustainable food purchases than having red-only ‘warning’ labels or green-only ‘low impact’ labels in real-world and online trials alike [15, 16]. These findings are supported by other work suggesting that red labels are more impactful on customer selections than green labels [17], and for environmental labels in particular [18]. Other work, albeit on pictorial warning labels, suggests negative labels on meat products may be effective at changing participant behaviour [19]. However, these studies are contrasted in part by an online supermarket study investigating the impact of labelling the top third (A-B carbon rating), top two-thirds (A-D carbon rating), or all products (A-E carbon rating), suggested labelling led participants to choose ‘lower CO_2_’ products, but no additive effect of further labelling beyond the top third of products [20]. However, the context of this study itself was relatively limited, with participants selecting a single ready meal, and there was a range of sometimes unlabelled products available throughout. However, no studies have yet determined whether changes in the range of a five-level traffic light composite ecolabel (e.g. ‘A’ – ‘E’) moderates their effect on sustainable food product selections.

Testing whether the range of ecolabels on offer moderates sustainable meal selection expands previous work on traffic light ecolabels and could help predict the contexts in which ecolabels might be most effective. By experimentally manipulating ranges of A – E ecolabels, we can investigate whether the extent to which previous findings observing no impact of ecolabels on behaviour [3] might be specific to contexts where limited ecolabel ranges may be on offer on a given day.

In addition, ecolabels might impact on business, rather than consumer behaviour, through encouraging caterers, restaurants and supermarkets to include options with lower environmental impact as part of their ranges [21, 22]. This increased product range might in turn enhance the impact of ecolabels on consumer behaviour, compared to what may be a narrow and more environmentally impactful range in some contexts currently. If this was the case, then results from the extant literature that suggest a limited impact of ecolabels but that explore only a narrow range of products, may not being providing a full picture of the potential for ecolabels to reduce the environmental impact of food.

As such, the current proof-of-principle study aimed to test whether smaller or larger ranges of ecolabels available in a food choice context alters the effectiveness of ecolabels on sustainable meal selections.

## Methods

### Overview

This study tested whether changes in the range of ecolabels on offer in a meal selection scenario moderated the effect of ecolabels on environmentally sustainable meal selection. The study protocol was pre-registered (https://osf.io/69xct/) and received ethics approval from the University of Oxford Central University Research Ethics Committee (CUREC Approval Reference: R85862/RE001). Hypotheses and the analyses plan were pre-specified before data collection.

### Participants

Participants were recruited from the online research platform Dynata (https://www.dynata.com/) via email. We recruited a sample of participants that reflected the UK population according to gender, age, and education status (based on the 2021 UK census). To take part, participants had to be aged 18 years or over, speak English, be a resident in the UK and provide informed consent. Participants that self-identified as vegan or vegetarian, or had other specific dietary restrictions, were excluded so that participants would be able to choose any meal in the study tasks. Participants were also excluded if they failed the attention check question (placed after the meal selection task), or if they completed the survey in less than 30% of the median completion time. Excluded participants and non-completers did not contribute to the demographic quotas.

### Study design

This study was a randomised controlled trial, which took place on the online survey platform Qualtrics (https://www.qualtrics.com/). Participants were randomised to one of two main study conditions: intervention (ecolabels present) or control (no ecolabels present). Participants assigned the intervention condition were repeatedly randomly assigned to one of nine ecolabel groups across five meal choice scenarios. Each intervention condition allocated a different combination of ecolabels to meals in a scenario (Table 1).

**Table 1:** Study conditions, with the ecolabels assigned to each low, medium and high impact meal

Study condition	Ecolabel group	Ecolabel ‘low’ impact meal	Ecolabel ‘medium’ impact meal	Ecolabel ‘high’ impact meal	Total range^1^
Intervention	ACE	A	C	E	4
Intervention	ACD	A	C	D	3
Intervention	BCE	B	C	E	3
Intervention	ACC	A	C	C	2
Intervention	BCD	B	C	D	2
Intervention	CCE	C	C	E	2
Intervention	BCC	B	C	C	1
Intervention	CCD	C	C	D	1
Intervention	CCC	C	C	C	0
Control	NA	None	None	None	NA

^1^Numerical score indicating breadth of ecolabels on offer in each meal selection scenario (while “range” can refer to the number and type of products available, here we use it to refer to the distance between the minimum and maximum values of the ecolabel scale). To calculate the “Total Range”, ecolabels were converted to numbers (A = 2, B = 1, C = 0, D = 1, E = 2) and were then summed for each study condition. For example, the Ecolabel Group “ACE” would score 2 + 0 + 2 = 4

To realistically manipulate the range of ecolabels on offer within a meal choice scenario, the environmental impact of each meal was first determined. The meal descriptions and images used in this study matched a meal that was offered in a UK cafeteria, for which an environmental impact score had been previously calculated (following methodology outlined in [3]). Each meal was then categorised as ‘low’, ‘medium’, or ‘high’ impact based on their comparative environmental impact to meals within their allocated meal choice scenario. Although meals were categorised by only comparing impacts within a given meal choice scenario, all meals in each impact category had similar environmental impact scores.

Each meal was then allocated a range of ecolabels that it could possibly be assigned, according to whether it was a ‘low’, ‘medium’ or ‘high’ impact meal. ‘Low’ impact meals (e.g. ‘Deli Veggie Burger’) could be assigned one of ‘A’, ‘B’ or ‘C’ ecolabels, ‘medium’ impact meals (e.g. ‘Hot Chicken Burger’) always scored ‘C’ and ‘high’ impact meals (e.g. ‘Spicy Beef Burger’) could score either a ‘C’, ‘D’ or ‘E’.

Each choice set contained one low, one medium and one high impact meal. Therefore, the range of ecolabels on offer could be manipulated for a given meal choice scenario (e.g. ‘Burgers’) to produce a maximum number of nine ecolabel groups with the medium-impact product always labelled ‘C’ (Table 1). In each meal choice scenario, only the ecolabels varied and the meals offered were kept constant.

### Ecolabels

**Fig. 1:**
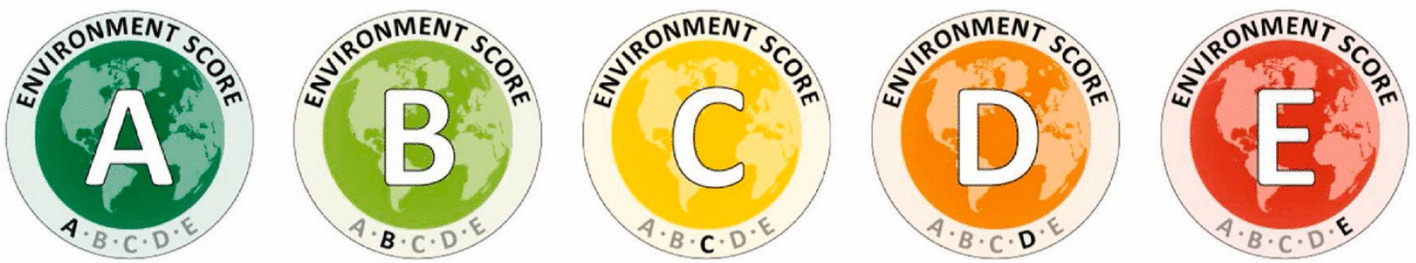
Ecolabels used in the study

Those assigned the control condition only saw an image and description of each meal choice, whilst those assigned the ecolabel group additionally saw an ecolabel adjacent to each meal choice (with identical ecolabel design to that used in [3]. The ecolabels had a letter and colour scaling system to indicate the overall environmental impact score of a product (Fig. 1: Green, ‘A’ labelled meals = lowest impact and red, ‘E’ labelled meals = highest impact).

### Study procedure

After giving informed consent, participants first completed questions on demographics (age, gender, education status, income), as well as meat eating frequency, and cafeteria attendance (see Supplementary Material 1.1 for survey questions).

Next, they completed a preference ranking task, ranking 15 hot meal options according to their meal preferences. All meals in the ranking task were included in the subsequent meal selection task. Participants then answered five unrelated image-based distractor questions before the main meal selection task.

**Fig. 2:**
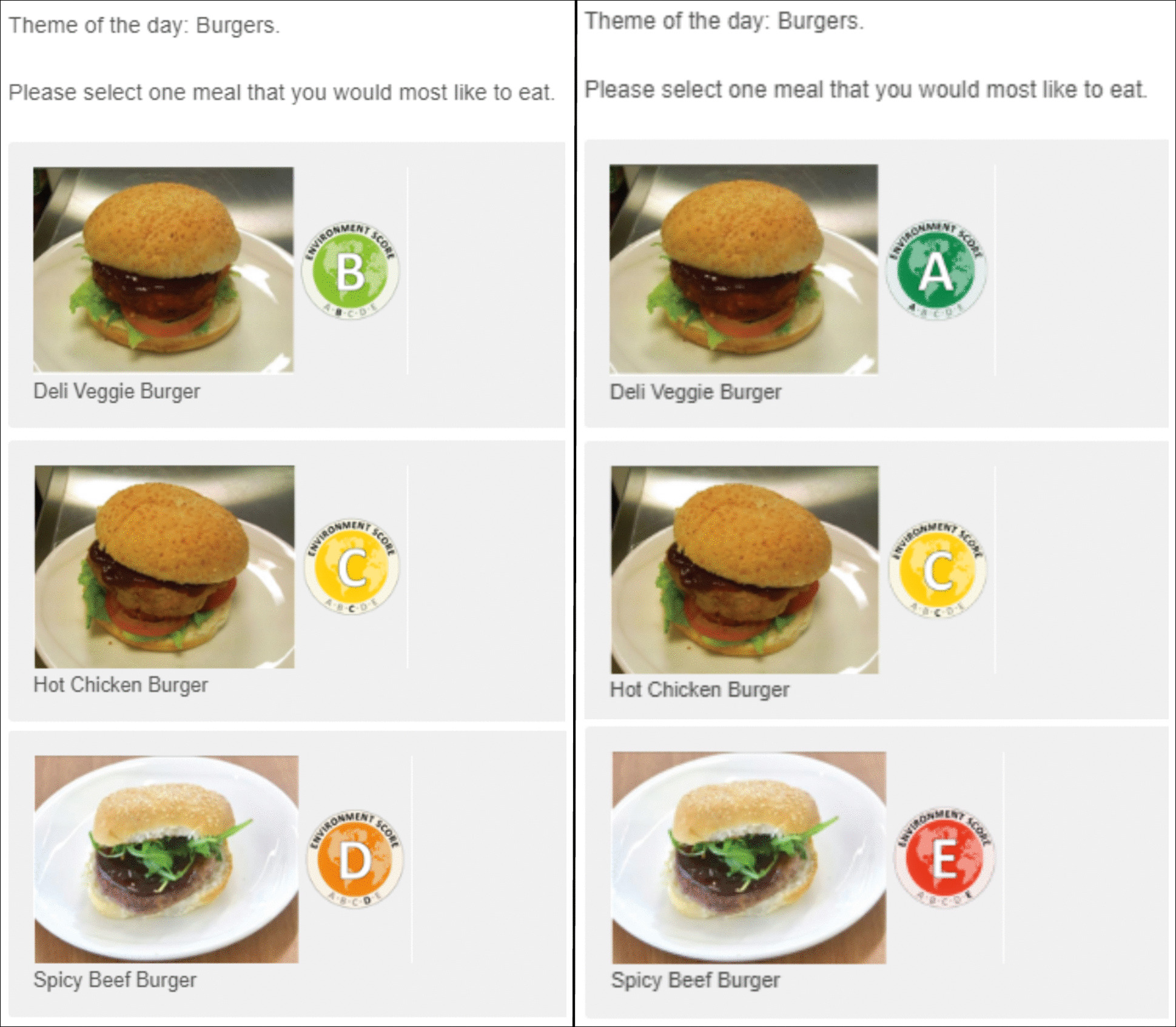
Example of what participants viewed in the meal selection task if they were assigned to either ‘Intervention: Ecolabel Group BCD’ (LHS) or ‘Intervention: Ecolabel Group ACE’ (RHS)

For the meal selection task, participants were presented with five meal selection scenarios in a random order (see Fig. 2 for an example scenario). Each meal selection scenario consisted of meals with a common theme (e.g. “Burgers”) and had three meals to choose from. In each question, participants were asked to select one meal they would most like to eat from the three options. The meal with the lowest environmental impact was always presented first, and the most environmentally impactful meal was always presented last.

### Sample size

Two power analyses were conducted in R to calculate the sample size needed, to account for the primary analysis (testing the effects of changing ecolabel range) and manipulation check (testing the effects of ecolabel vs no ecolabel). In the absence of appropriate previous literature to help us determine a more precise effect size for the primary analyses, we powered to detect a very small effect size (d = 0.1) [23]. Therefore, for comparing differences between ecolabel range conditions: a minimum sample size of 257 participants per ecolabel group, 2310 in total, was required (accounting for a small effect size d = 0.1, power of 95%, alpha = 0.05, and 5% attrition). For comparing intervention (ecolabel) groups vs. control: an additional 322 participants were required (accounting for a medium effect size d = 0.27 [24], a power of 95%, alpha = 0.05, and 5% attrition). In total a sample size of 2,632 was required.

### Statistical analyses

We completed a series of analyses in R studio [25] using packages sjstats, lme4 and DHARMa [26–28]. Details of variables and participant characteristics used in the analyses are outlined in Supplementary Material 1.3 and 1.4.

#### Primary analyses

The primary analyses investigated the question: *Does the size of ecolabel range on offer impact selections of more sustainable meal options*? The two binary primary outcome measures used to test this question are based on responses to the meal selection task.

- For the outcome measure *Low*, a participant scored 1 if they selected the lowest impact meal, and 0 if they selected the medium or high impact meal in a meal selection scenario.
- For the outcome measure *Low/Medium*, a participant scored 1 if they selected the lowest or medium impact meal, or scored 0 if they selected a high impact meal.

As such, the outcome measures *Low* and *Low/Medium* test whether the changing ecolabel ranges shifted participant meal selections towards the lowest impact meal, and/or away from the highest impact meal.

To test for the effects of different ecolabel ranges, we created the variable Total Range, which represents the breadth of ecolabels on offer in each meal selection scenario (Table 2). To calculate the Total Range, ecolabels were converted to numbers (A = 2, B = 1, C = 0, D = 1, E = 2) and were then summed for each study condition. For example, the Ecolabel Group “ACE” would score 2 + 0 + 2 = 4.

To compare the effects of the Total Range on the primary outcome measures, we constructed binomial generalised linear mixed models (GLMMs), with random effects by participant. Results from the control group (no ecolabels) were not entered into the models, as we compared the relative effectiveness of different ecolabel ranges. Relevant participant characteristics (age, gender, household income, education status, and the device the study was completed on) were included to account for between participant variation.

#### Manipulation check

The manipulation check investigated the question: *does the presence of ecolabels change meal selections compared to the control*? To answer this question, two outcome measures were analysed: *Low score* and *Low/Medium score*. Respectively, these outcome measures are the sum of the *Low* or *Low/Medium* primary outcome measure per person. For example, if a participant in total selects three low impact meals, one medium impact meal, and one high impact meal, then their *Low score* would be three, and their *Low/Medium score* would be four.

To compare the effects of the ecolabel (all intervention groups combined) vs control (no labels) on *Low score* and *Low/Medium score*, we constructed ordinal logistic regression models.

#### Deviations from protocol

Both the primary and manipulation check outcome measures differ from the pre-specified outcome measures, which relied upon participants’ responses to the preference ranking task. However, there were concerns about inaccurate completion of this ranking task—in particular, the results did not align with participants’ meal scenario choices. Therefore, the outcome measures were based upon results from the meal selection task only. Supporting evidence for this decision is provided in Supplementary Material 1.5.

As there are twice the number of outcome variables than powered for, we halved the pre-specified Bonferroni-adjusted threshold of significance, such that *p* < 0.025 determined the statistical significance for models in primary analyses. We used a threshold of *p* < 0.01 to determine statistical significance for the manipulation check.

In addition, we did not complete a pre-specified sensitivity analysis, and additional secondary analysis, as each depended on the completion of the ranking task by participants. Other pre-specified secondary analyses (two GLMMs to test for effects of positive and negative Ranges on the primary outcome variables, accounting for relevant participant characteristics) were run for completeness (Supplementary Material 2.3), although their interpretation is now limited by the change in outcome measure.

## Results

### Summary of participants

**Fig. 3:**
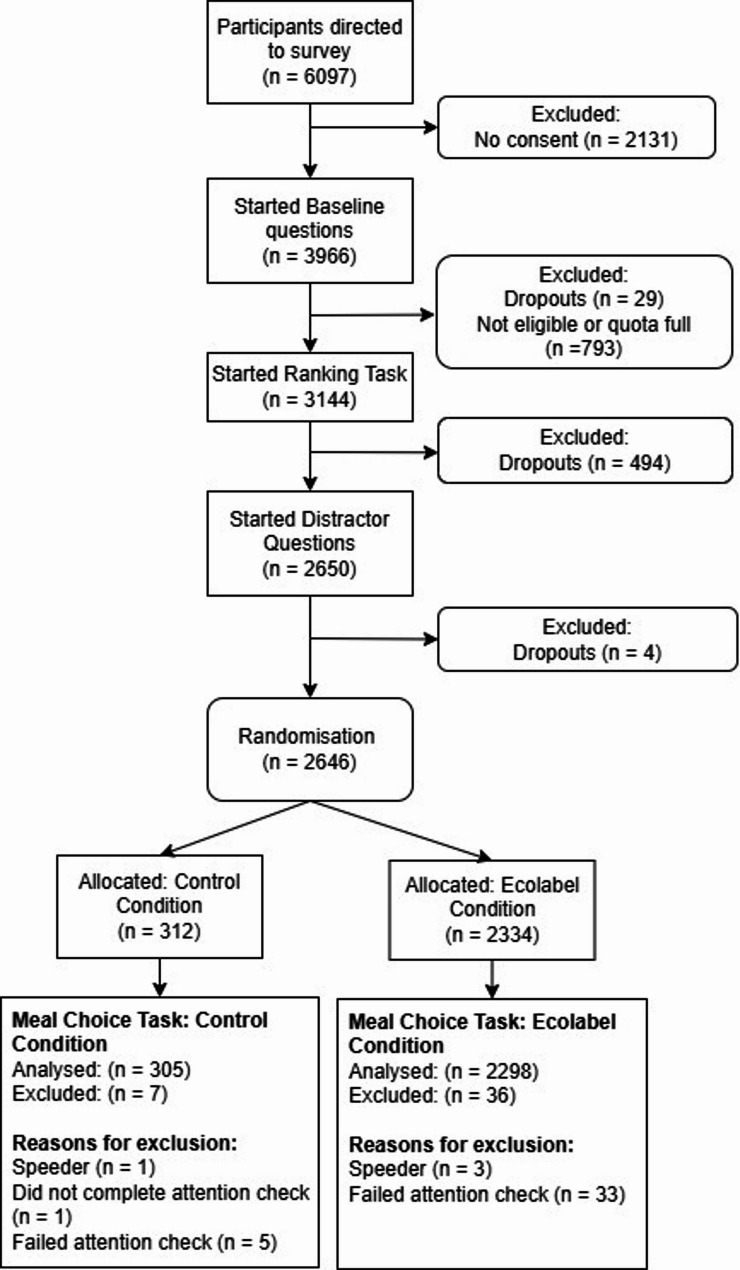
Study flow diagram showing the number of participants that were excluded from analyses at each stage, which a brief reason for exclusion

Of the participants directed to the survey, a total of 3451 participants were excluded before they were allocated to a condition (Fig. 3). Most of the excluded participants (*n* = 2131) were removed because they did not provide consent by ticking both boxes certifying that they were above 18 and that they agreed to take part. In the meal selection task, 312 participants were randomised to the control condition, of which 98% (*n* = 305) completed the study and 2334 were randomised to the ecolabel condition, of which 98% (*n* = 2298) completed the study (Fig. 3).

The participants completing the study were broadly representative of the UK population in terms of age and gender (Table 2). There were some differences between the sample and the UK population in the education category – for example, only 5% of the sample had “no education”, whilst 18.2% of the UK population fall into this category. Most participants reported that they ate meat at least 3–4 days a week, and approximately a third reported that they never purchase breakfast, lunch or dinner from canteens or cafeterias (Table 2).

**Table 2:** Demographic breakdown of all participants that completed the survey (*n* = 2603), by age, education status, gender, household income, frequency of going a cafeteria, and total meat-eating frequency score. The demographic breakdown of the UK population in 2021 (based on the 2021 UK Census) is provided for age, education status, and gender

**Age**	N	Percentage of sample (%)	Percentage of UK population (%)
18–34	710	27.3	27.6
35–49	632	24.3	24.4
50–64	648	24.9	24.6
65 +	613	23.5	23.4
**Education Status**^1^	N	Percentage of sample (%)	Percentage of UK population (%)
Apprenticeship	66	2.5	5.3
Level 1 and entry level qualifications	454	17.4	9.6
Level 2 qualifications	544	20.9	13.4
Level 3 qualifications	422	16.2	16.9
Level 4 qualifications or above	950	36.5	33.8
No qualifications	131	5.0	18.2
Other	36	1.4	2.8
**Gender**	N	Percentage of sample (%)	Percentage of UK population (%)
Female	1339	51.4	51.6
Male	1259	48.4	48.4
Prefer to self-identify	5	0.2	Not Recorded
**Household income**	N	Percentage of sample (%)	
Less than £25 K	711	27.3	
Between £25 K and £39 K	787	30.2	
£40 K or above	988	38.0	
Prefer not to say	117	4.5	
**Frequency of going to a cafeteria to buy breakfast, lunch, or dinner**	N	Percentage of sample (%)	
1–2 days a week	447	17.2	
3–4 days a week	200	7.7	
5–6 days a week	48	1.8	
Every day of the week	17	0.7	
Less than once a week	922	35.4	
Never	952	36.6	
Prefer not to say	15	0.6	
Did not answer	2	0.1	
	Mean	Standard Deviation	
**Total Meat-Eating Frequency score**^2^	5.6	2.0	

^1^Education status

Level 1 and entry level qualifications: 1 to 4 GCSEs grade A* to C, Any GCSEs at other grades, O levels or CSEs (any grades), 1 AS level, NVQ level 1, Foundation GNVQ, Basic or Essential Skills

Level 2 qualifications: 5 or more GCSEs (A* to C or 9 to 4), O levels (passes), CSEs (grade 1), School Certification, 1 A level, 2 to 3 AS levels, VCEs, Intermediate or Higher Diploma, Welsh Baccalaureate Intermediate Diploma, NVQ level 2, Intermediate GNVQ, City and Guilds Craft, BTEC First or General Diploma, RSA Diploma

Level 3 qualifications: 2 or more A levels or VCEs, 4 or more AS levels, Higher School Certificate, Progression or Advanced Diploma, Welsh Baccalaureate Advance Diploma, NVQ level 3; Advanced GNVQ, City and Guilds Advanced Craft, ONC, OND, BTEC National, RSA Advanced Diploma

Level 4 qualifications or above: degree (BA, BSc), higher degree (MA, PhD, PGCE), NVQ level 4 to 5, HNC, HND, RSA Higher Diploma, BTEC Higher level, professional qualifications (for example, teaching, nursing, accountancy)

^2^Aggregate score (from 0 to 10) of meat eating frequency at lunch and dinner: whereby answers for separate lunch and dinner questions were scored ‘Never’ (0), ‘Less than once a week’ (1), ‘1–2 days a week’ (2), ‘3–4 days a week’ (3), ‘5–6 days a week’ (4), ‘Every day of the week’ (5)

### Summary of meal selections

**Fig. 4:**
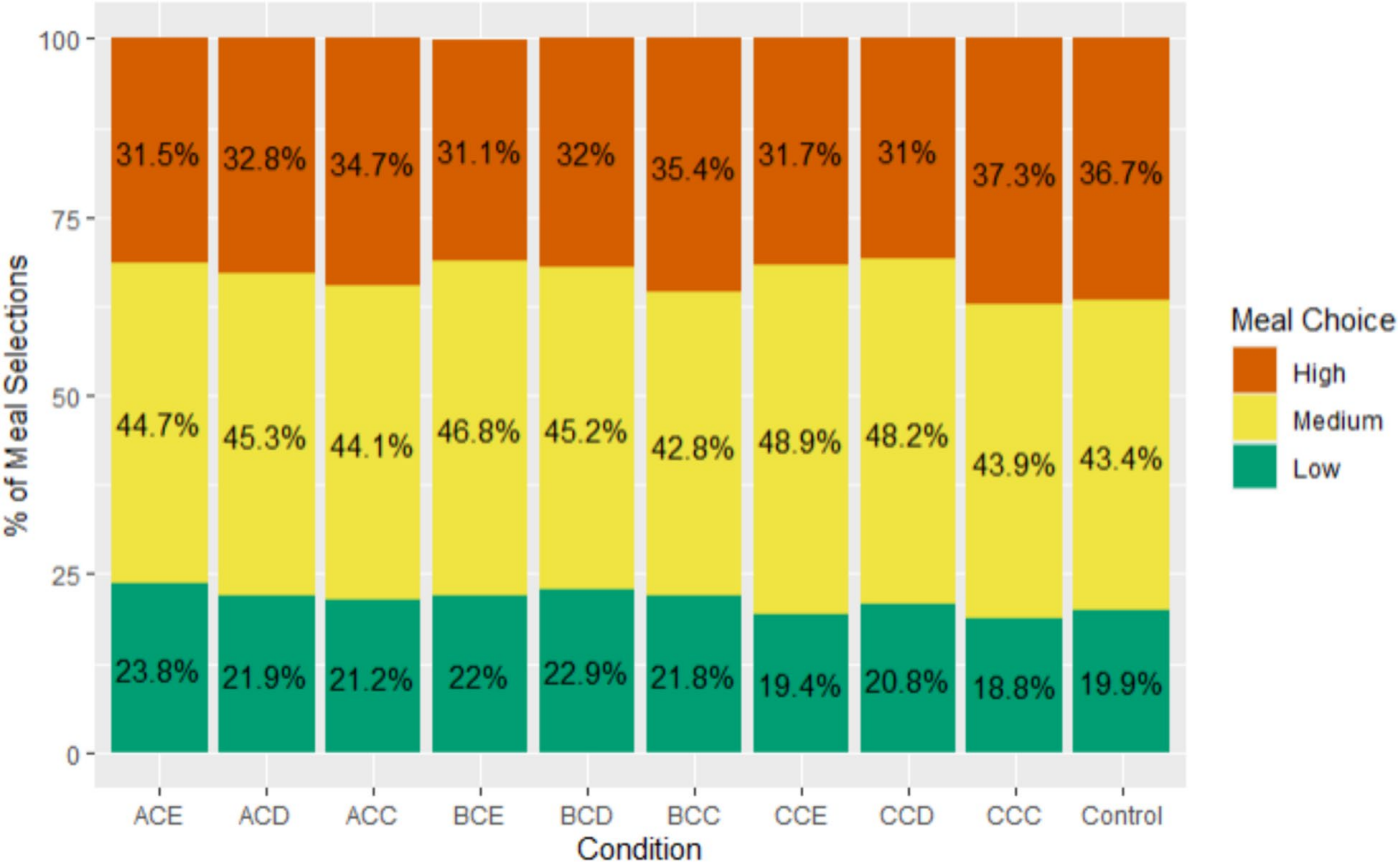
Stacked bar graph indicating the percentage of selections that were low, medium and high impact meals in the meal selection task, grouped by the ecolabels presented in a meal choice to participants, and the control group

In total, 13,015 meal selections were made in the meal selection task (2603 participants making five selections each). In the control group, 19.9% (*n* = 303) of meal selections were ‘low’, 43.4% (*n* = 662) were ‘medium’, and 36.7% (*n* = 560) were ‘high’ impact meals. Across all the ecolabel groups, 21.4% (*n* = 2460) of participants’ meal selections were ‘low’, 45.5% (*n* = 5232) were ‘medium’ and 33.1% (*n* = 3798) were ‘high’ impact meals. Meal selections grouped by the ecolabels presented in a meal selection scenario are summarised in Fig. 4. Participants who saw the largest range of ecolabels (ACE) chose 23.3% low impact meals and 31.5% high impact meals, compared to 18.8% low impact and 37.3% high impact meals for those who saw CCC only (Fig. 4).

### Manipulation check: did ecolabels change meal selection?

When presented with ecolabels, participants had significantly greater odds of choosing a Low or Medium impact meal than those not presented with ecolabels (OR: 1.34, 95% CI: 1.09–1.67, *p* = 0.007). However, those presented with ecolabels did not have significantly greater odds of choosing the Low impact meal in comparison to the control group (OR: 1.19, 95% CI: 0.95–1.48, *p* = 0.13).

### Primary analysis: effects of total range on meal selection

Participants presented with the smallest range of ecolabels (CCC, Total Range = 0) had significantly lower odds of choosing a *Low/Medium* or *Low* impact meal than participants presented with a largest range (ACE, Total Range = 4) (see Table 3). Participants presented with a small range of ecolabels (BCC or CCD, Total Range = 1) also have lowered odds of choosing a *Low* impact meal than those presented with the largest range (ACE), however this difference was non-significant (*p* = 0.028). Overall, once the Total Range was greater than 0, there was no statistically significant evidence increasing it further affected meal selection.

**Table 3:** The effect of Total range on the selection of low/medium impact meals (LHS) and the selection of Low impact meal (RHS)

*Predictors*	*Outcome measure*					
	*Low/medium*			*Low*		
	*Odds ratios*	*CI*	*P*	*Odds ratios*	*CI*	*p*
(Intercept)^1^	3.59	2.98–4.33	** < 0.001**	0.24	0.19–0.31	**< 0.001**
Total Range [0]	0.77	0.65–0.91	**0.003**	0.71	0.58–0.88	**0.001**
Total Range [1]	0.92	0.79–1.06	0.244	0.82	0.69–0.98	0.028
Total Range [2]	0.94	0.82–1.08	0.400	0.84	0.71–1.00	0.045
Total Range [3]	0.98	0.85–1.14	0.828	0.88	0.74–1.05	0.151
Random Effects						
σ^2^	3.29			3.29		
τ_00 ResponseId_	0.09			0.65		
ICC	0.03			0.17		
Marginal R^2^/Conditional R^2^	0.031/0.058			0.038/0.197		

^1^Both model intercepts are set at: Total Range = 4, Age = 18–34 years, Gender = Female, Education Status = Level 1 Qualifications, Household Income = More than £40k, Device = Mobile. Significant p-values (p < 0.025) are highlighted by bold text. Predicted probabilities are reported in Supplementary Material 2.1 for this model

### Exploratory analyses: interactions between participant characteristics, meat eating or canteen attendance and total range

There were no significant interaction effects between Total Range and gender, household income, meat eating frequency, frequency of participants’ cafeteria visits, or device used on selection of Low/Medium or the Low meal. There were no consistent patterns in interaction analyses between Total Range and age, and Total Range and education status on selection of Low or Low/Medium meals (Supplementary Material 2.5).

## Discussion

This large online study demonstrates that altering the range of ecolabels on offer to participants can moderate the effect of ecolabels on sustainable meal selection. We find that presenting any range of ecolabels in a meal choice scenario encourages sustainable meal selection more than no range (e.g. ‘CCC’), but limited evidence of impact between other ranges (e.g. ‘BCC’ vs ‘ACC’). In addition, analysis aggregating across all ecolabels suggested these discouraged the selection of high impact meals, compared to when no labels were present.

Results from this study’s comparison of ecolabels against no labels adds further evidence that ecolabels encourage sustainable meal selections. This finding is consistent with systematic reviews, such as Potter et al., [8], and similar online trials of coloured ecolabels [24, 29], which suggest that A-E ecolabelling could be effective at encouraging people to reduce the environmental impact of their food purchases. However, this effect has not been detected when the same ‘A’—‘E’ ecolabels were trialled in UK worksite cafeterias [3, 38]. The primary purpose of this study was to explore how individuals respond to different combinations of ecolabels, which can provide insights into the results of existing ecolabel field trials. For example, the evidence from in the current study suggests that Pechey et al.’s [3] finding that ecolabels have no effect on sustainable meal purchasing may have been impacted by very limited ecolabel ranges available on given days. However, a wider ecolabel range may be likely in many other food purchasing settings such as supermarkets.

More specifically, we find that ecolabels may be more successful at encouraging participants to choose fewer ‘D’ or ‘E’ ecolabelled meals, than to choose more ‘A’ or ‘B’ meals. This ties in with previous research suggesting that red labels may have greater impact than green labels [17] and negative labels more than positive ones [30], perhaps due to loss aversion biases [31]. Yet this result contrasts with findings from Chandon et al.’s [20] study on carbon labelling for frozen meals, which found labelling only ‘low’ impact meals was sufficient to encourage participants to choose more sustainable meal options. Additional labelling of ‘medium’ or ‘high’ impact meals did not provide any significant improvement to the sustainable food choices. Further work is needed on real world selections across multiple items. If ecolabels discourage the selection of the most impactful food products, this may be particularly beneficial for reducing environmental impact, given shifting consumer selections away from ‘D’/’E’ to ‘C’ could have a bigger environmental impact saving than switching from a ‘C’ to ‘B’/’A’ labelled meal, due to the strong positive skew of the environmental impacts of food [2]. The study results may also be used to consider the likely relative impact of ecolabels in contexts where menus have broader or narrower ranges of food products available.

To our best knowledge, this study offers a novel test of the effect of ecolabel ranges on food selection. Particular strengths of this study include the randomised control design, use of a sample of the UK population that reflected key demographic characteristics, and realistic food choice scenarios. However, as the study was hypothetical in design, participants did not receive the meals they selected, so may be less inclined to choose the meal they actually preferred, and, due to concerns regarding the accuracy of the ranking task (further explained in Supplementary Material 1.5) our analysis could not account for participants’ underlying meal preferences. Future online studies could limit the number of meal choices to be ranked, reducing the effort required to rank options, increasing the likelihood that the task is completed properly. We note that removing the ranking task and subsequently changing the primary outcome from a single measure to two co-primary outcomes may have increased the likelihood of type II errors due to the adjustment of the significance threshold from *p* = 0.05 to *p* = 0.025. However, this Bonferroni correction was necessary to account for the two co-primary outcomes and to avoid an increased risk of type I errors.

Additionally, the ecolabels in this study are likely to be much more salient than ecolabels in real world settings, as there are fewer distractions (such as nutritional labelling). As participants were aware that their choices were being monitored for a study, they may be more likely to choose lower impact meals due to social desirability bias [32]. As such, the effect size of ecolabels found here may be larger than that found in real-world settings, as found in comparisons between hypothetical and real-world food labelling studies [33]. However, this effect is minimised for the ecolabel range comparisons, given the presence of ecolabels in each group. Finally, in the meal selection task, ‘low’ impact dishes were always presented first and ‘high’ impact dish always last. This may have caused confounding effects of ordering (whereby the option presented first is most frequently chosen) and sequential effects (whereby decisions are influenced by recent prior choices) [34, 35]. To avoid this, future studies could randomise the presentation of meals in selection tasks. However, any potential confounding effects were limited in this study, given we examined relative effects between conditions, and all participants were exposed to the same order of choices across all conditions.

Whilst the meal descriptions and images were realistic, the ecolabels assigned to each meal were only broadly reflective of their environmental impact. For example, a ‘low’ impact meal could be assigned a ‘A’, ‘B’ or ‘C’ ecolabel. If participants were aware of the environmental impact of different food groups, they might have been surprised by the allocation of ecolabels to meal types, which may have affected their meal choices. For example, some ecolabel combinations may be unlikely to occur in real-world settings – e.g. when Deli Veggie Burger, Hot Chicken Burger and Spicy Beef Burger are all labelled ‘C’. In these cases, if participants were aware of the appropriate ecolabel for each meal, they may have suspected manipulation, which may have altered their response to the labels. However, manipulating ecolabels rather than changing meals, ensured that any differences observed between ecolabel conditions were not due to the presence or absence of particular meal options.

Future studies could explore potential mechanisms underlying any effects of ecolabels and any moderators, e.g. exploring the role of consumer perceptions of meal sustainability with meal selections under different range conditions. For example, in this study, ecolabels could have updated pre-existing participant perceptions of the relative sustainability of meal options. If all options in a meal selection scenario scored a ‘C’ ecolabel, then this may have changed participant perceptions on the sustainability of meals (e.g. these scores suggest a beef burger is as sustainable as a vegetarian burger), and thus participants may have changed their selections accordingly.

In addition, future studies could investigate consumer perceptions of ‘A’ – ‘E’ ecolabels to contextualise study findings. For example, participants could be asked about their trust of ‘A’ – ‘E’ ecolabels to accurately represent the true environmental impact of the dishes. If participants do not find the matches of dishes and meals credible, then they may not change their selections accordingly, even if they have pro-environmental intentions and attitudes. Theoretical studies have highlighted the critical role of ecolabel credibility on pro-environmental consumer behaviour [36, 37], but the relationship has not yet been investigated for ‘A’ – ‘E’ traffic light ecolabelling of meals. If participants do find ecolabels credible, future research could explore whether ecolabels modify the extent to which consumers are willing to substitute their meal preferences with more sustainable options. Specifically, research could test whether there are “ecolabel tipping points” that need to be exceeded for consumers to make sustainable switches. For example, studies could determine the minimum distance in ecolabel score required for a consumer to willingly substitute their preference meal with a lower impact and lower preference meal. This would build upon the current study’s finding that offering meals labelled ‘ACE’ encourages more sustainable choices than having meals labelled ‘CCC’, with similar patterning, albeit non-significant, for those labelled ‘CCD’ or ‘BCC’. Further, scales with a larger range of values (e.g. ‘A’ to ‘G’, as opposed to ‘A’ to ‘E’) may be beneficial at increasing the potential distance between values, and this could be explored in further research. Overall, moderators of ecolabel effectiveness need further investigation, although our findings suggest that ecolabel range is of limited importance for ecolabels to effectively encourage sustainable meal selection, as long as more than one ecolabel score is on offer.

In terms of practical implications, where ecolabels are in use in contexts where limited ranges of options are likely such as in canteens or restaurants, results suggest adding or reformulating to make 'A'-labelled meals consistently offered (if they aren’t already) may impact sales beyond the impact expected simply from changing availability. Whilst adding ‘E’ meals on days where they are not already available would further increase the range on a given day, this would clearly not be recommended. It would also be unlikely to reflect how ecolabels have the potential to not only shift consumer behaviour, but also corporate behaviour. For example, the implementation of ecolabels could encourage restaurants, cafeterias or supermarkets to increase the number of environmentally sustainable products they offer. This theory of change is supported by studies of other types of environmental and health labelling interventions, which find that the introduction of such labels can encourage food providers to change the food they offer so that they are more healthy or sustainable [21, 22].

## Conclusions

Our findings support previous research indicating that ecolabels can increase the sustainability of food selections and expands on these to suggest that offering products with a wider range of ecolabel values is more effective than offering products with a narrower range of values. Our results have strong evidence for the widest vs. narrowest ranges, while there is more limited evidence currently on the impact of ecolabels when intermediate ranges are present.

## Supplementary Information

Supplementary Material 1.

## Data Availability

Anonymised survey results are available on the Open Science Framework: https://osf.io/69xct/files/a5xts.
