# Supplementary material for: Does the range of ecolabels on offer alter their impact on meal selection? An online randomised control trial
Source: BMC Public Health. 2025 Dec 13;26:226. doi: 10.1186/s12889-025-25870-8 (PMC12817769; doi:10.1186/s12889-025-25870-8)
Supplement: Supplementary file 1 — Supplementary Material 1. [file 12889_2025_25870_MOESM1_ESM.docx]

Supplementary Material

Contents

[1. Methods 2](#_Toc153398257)

[1.1 Survey Questions 2](#_Toc153398258)

[1.2 Meal types available in the meal selection task 10](#_Toc153398259)

[1.3 Model Variables 10](#_Toc153398260)

[1.4 Participant Characteristics 11](#_Toc153398261)

[1.5 Deviations from protocol 12](#_Toc153398262)

[2. Results 15](#_Toc153398263)

[2.1 Primary Analysis – full table with demographics 15](#_Toc153398264)

[2.2 Primary Analysis – full table with demographics and intercept Range = 0 16](#_Toc153398265)

[2.3 Secondary Analysis 17](#_Toc153398266)

[2.4 Effects of Participant Characteristics on meal selection 18](#_Toc153398267)

[2.5 Models Including Interactions between Participant Characteristics and Ecolabel range 18](#_Toc153398268)

## Methods

### 1.1 Survey Questions

#### Screening Questions

|  |  |
| --- | --- |

Q1 Are you:

- Under 18 years old?
- 18 years old or over?

Q2 Are you fluent in English?

- Yes
- No

Q3 Do you currently live in the UK?

- Yes
- No

Q4 Do you have any dietary restrictions?

- Vegan
- Vegetarian
- Gluten-free
- Sugar-free
- Dairy/lactose-free
- None
- Other (please specify below)

#### Attentional Check Question WARNING

Attentional Check Thank you for consenting to take part in this study.

To check that we have your full attention throughout the study, we have placed one simple and easy question in this study. If you do not answer this question correctly, you will be withdrawn from the study, redirected to Dynata and will not be reimbursed for your time.

#### Baseline Survey 1

Q5 Please indicate what gender you identify with.

- Male
- Female
- Prefer to self-identify __________________________________________________

Q6 Please select your age group.

- 18-34
- 35-49
- 50-64
- 65+

Q7 What is the highest education qualification you have achieved?

- **No qualifications**
- **Level 1 and entry level qualifications**: 1 to 4 GCSEs grade A* to C , Any GCSEs at other grades, O levels or CSEs (any grades), 1 AS level, NVQ level 1, Foundation GNVQ, Basic or Essential Skills
- **Level 2 qualifications**: 5 or more GCSEs (A* to C or 9 to 4), O levels (passes), CSEs (grade 1), School Certification, 1 A level, 2 to 3 AS levels, VCEs, Intermediate or Higher Diploma, Welsh Baccalaureate Intermediate Diploma, NVQ level 2, Intermediate GNVQ, City and Guilds Craft, BTEC First or General Diploma, RSA Diploma
- **Apprenticeship**
- **Level 3 qualifications**: 2 or more A levels or VCEs, 4 or more AS levels, Higher School Certificate, Progression or Advanced Diploma, Welsh Baccalaureate Advance Diploma, NVQ level 3; Advanced GNVQ, City and Guilds Advanced Craft, ONC, OND, BTEC National, RSA Advanced Diploma
- **Level 4 qualifications or above**: degree (BA, BSc), higher degree (MA, PhD, PGCE), NVQ level 4 to 5, HNC, HND, RSA Higher Diploma, BTEC Higher level, professional qualifications (for example, teaching, nursing, accountancy)
- **Other**: vocational or work-related qualifications, other qualifications achieved in England or Wales, qualifications achieved outside England or Wales (equivalent not stated or unknown)

#### Preference Ranking Task

Please imagine that you are choosing lunchtime meals for you to eat in a workplace canteen.
Please order the following meals according to what you would most like to eat from a workplace canteen (1st place), to what you would least like to eat from a workplace canteen (15th place).

Please **click and drag** each option to rank each meal option from 1-15.

**1st place** = the meal you would **most** like to eat
**15th place** = the meal you would **least** like to eat

| Deli Veggie Burger | 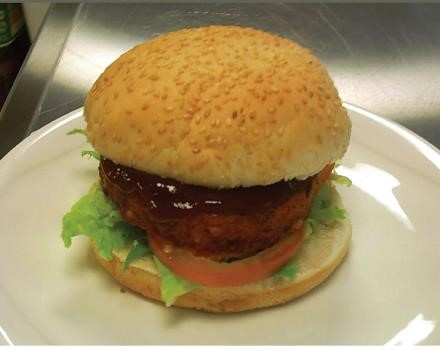 |
| --- | --- |
| Hot Chicken Burger | 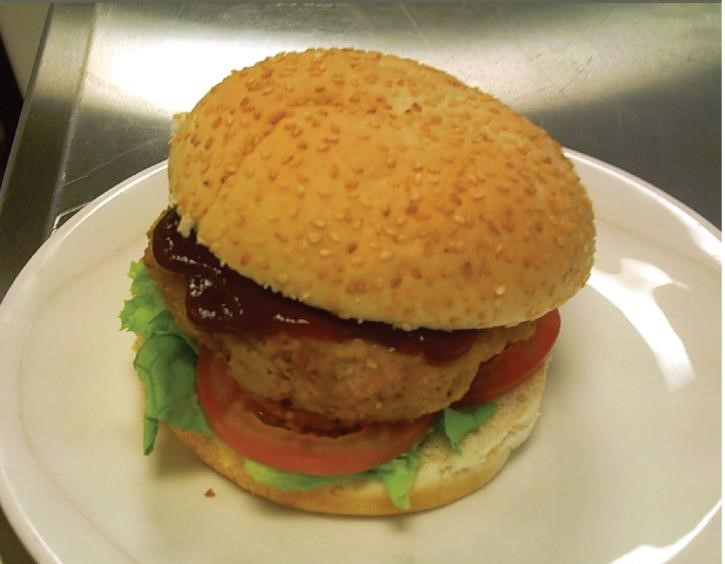 |
| Spicy Beef Burger | 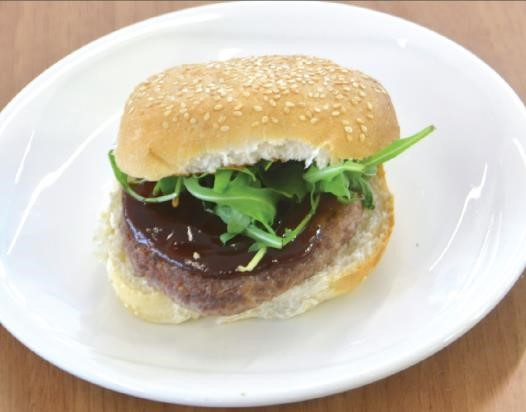 |
| Chicken Sweetcorn Pasta Bake | 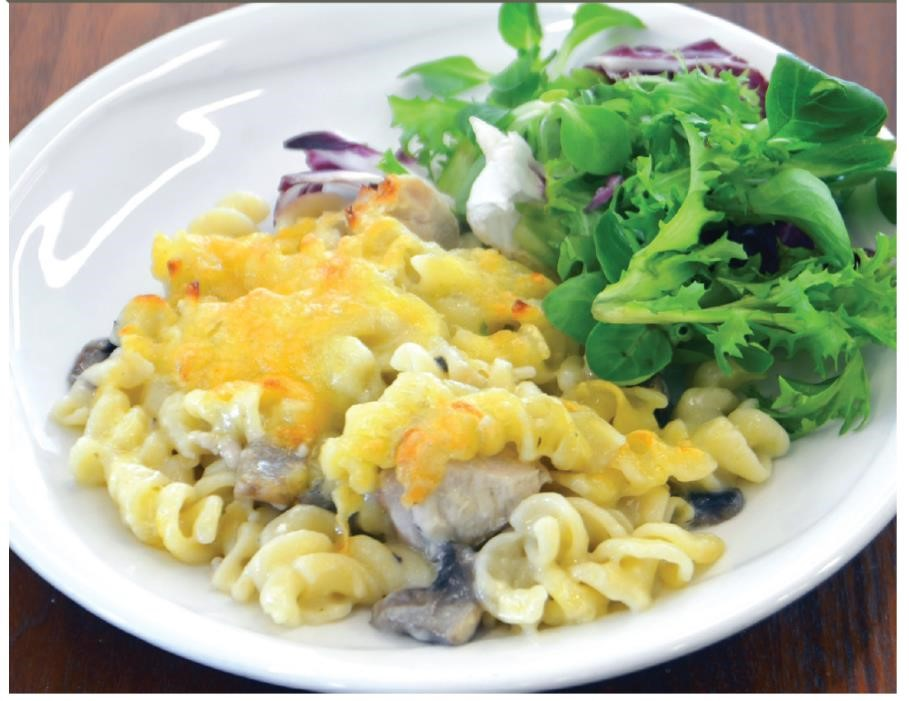 |
| Macaroni Cheese | 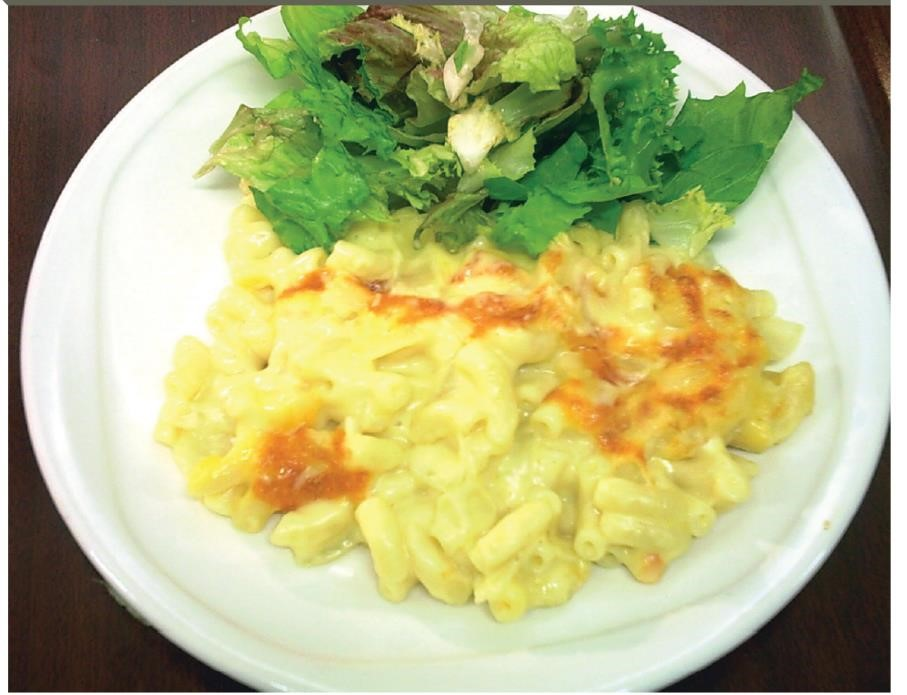 |
| Beef Lasagne | 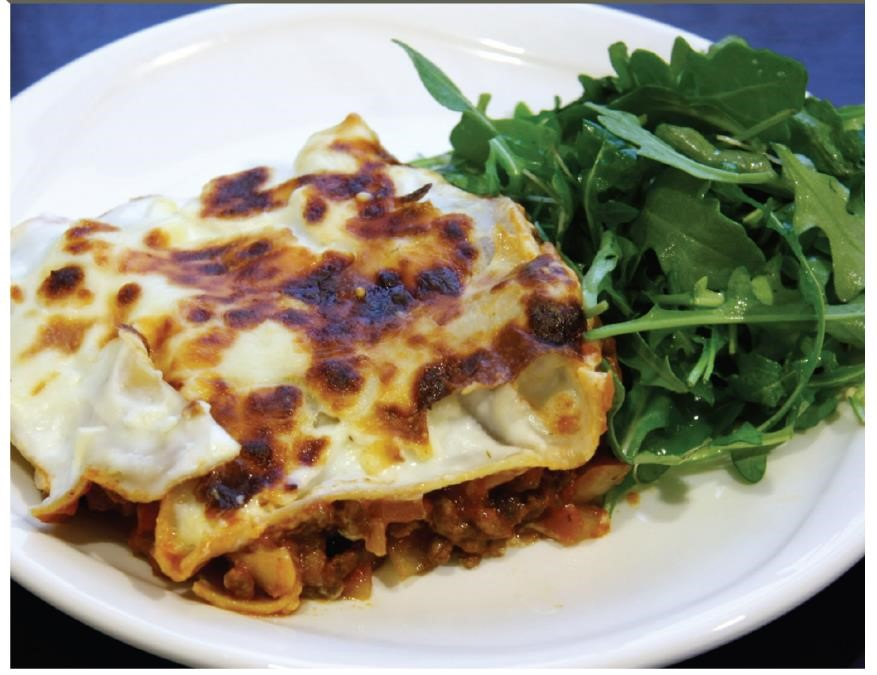 |
| Vegetable Masala Curry | 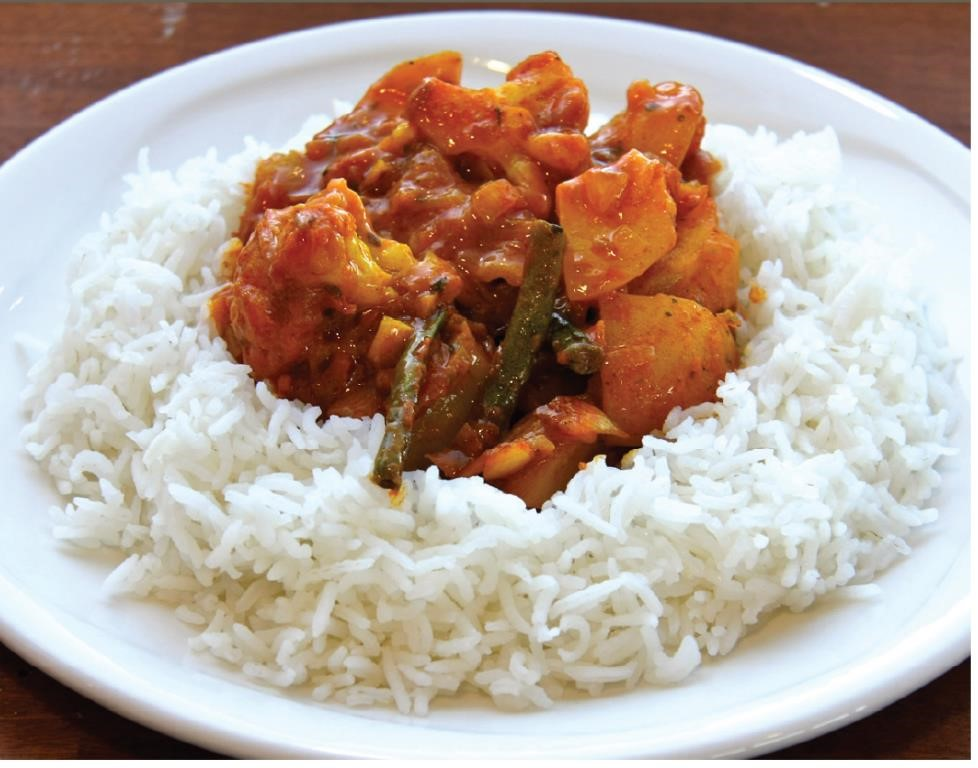 |
| Chicken Balti Curry | 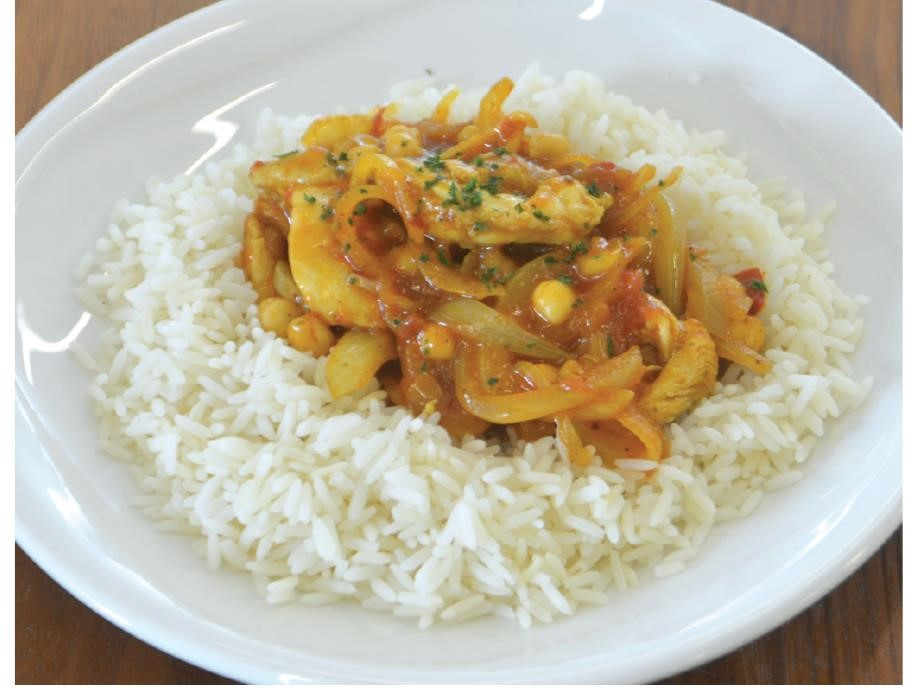 |
| Beef Madras Curry | 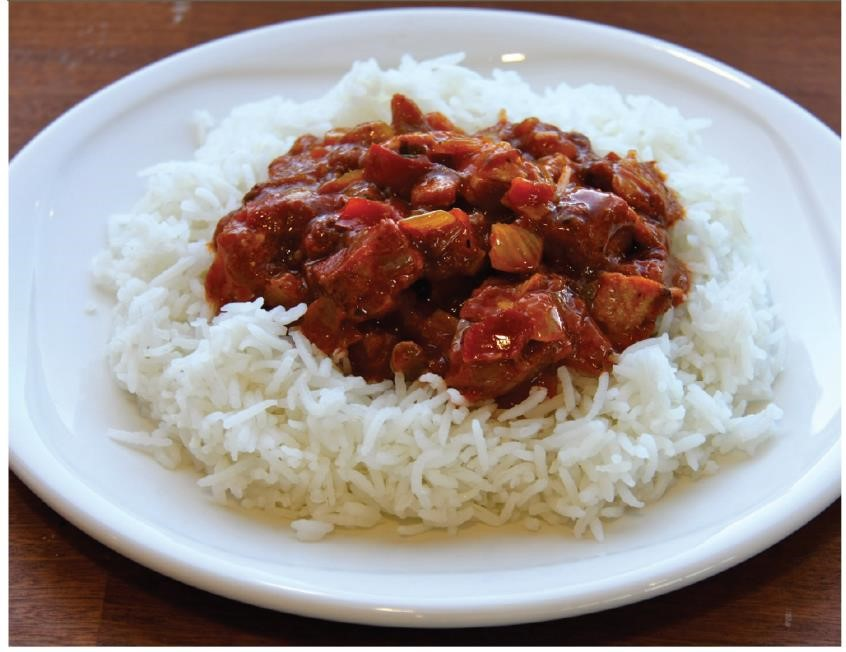 |
| Vegetable Thai Green Curry | 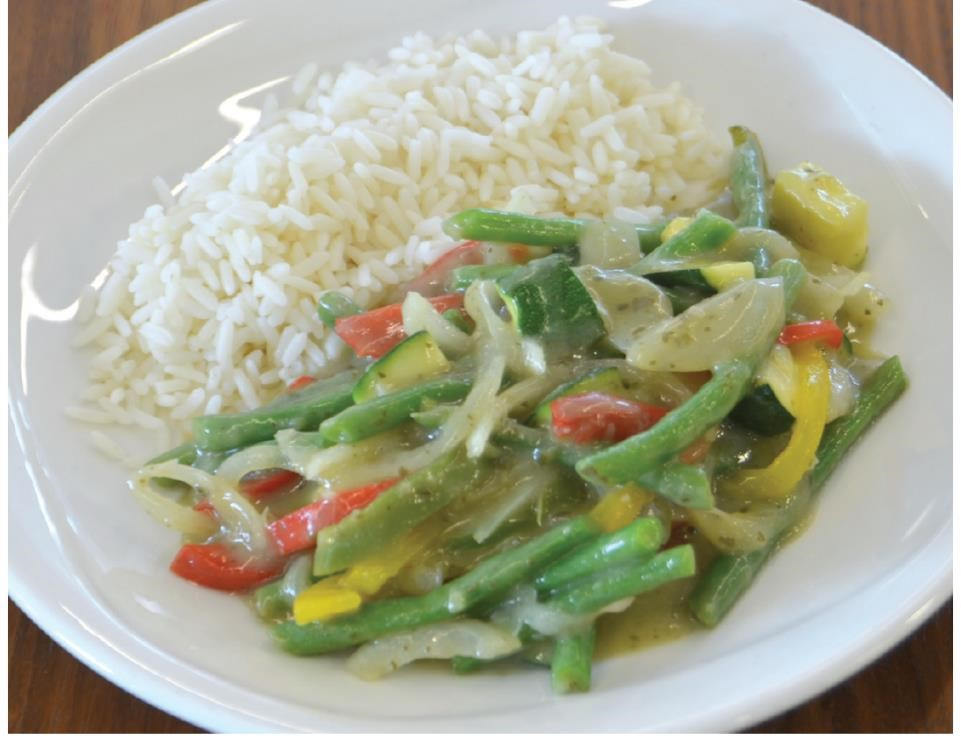 |
| Chicken Thai Red Curry | 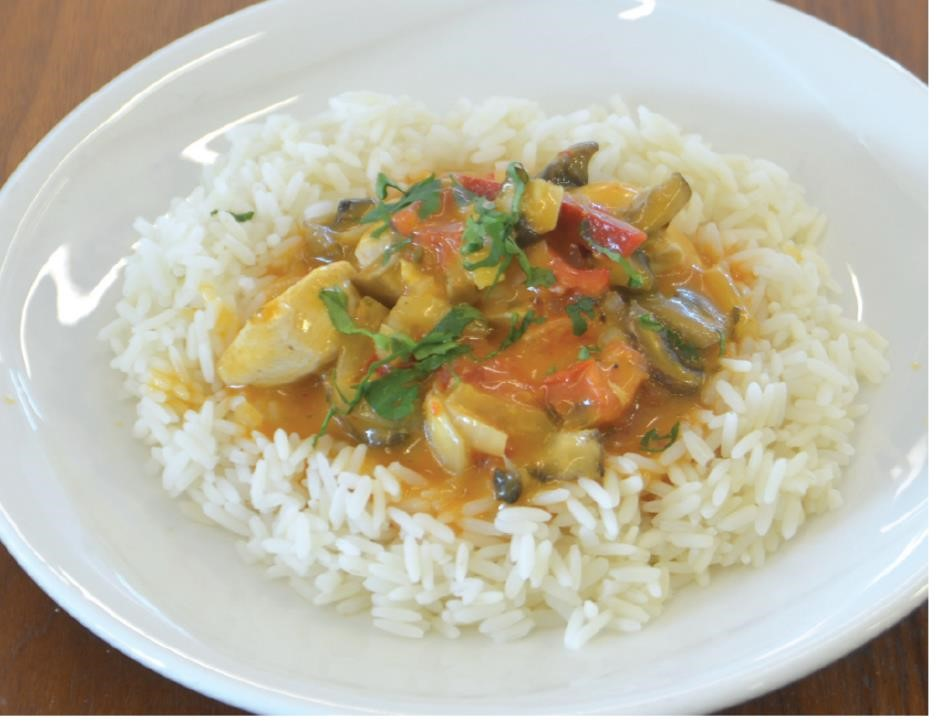 |
| Sweet and Sour Pork | 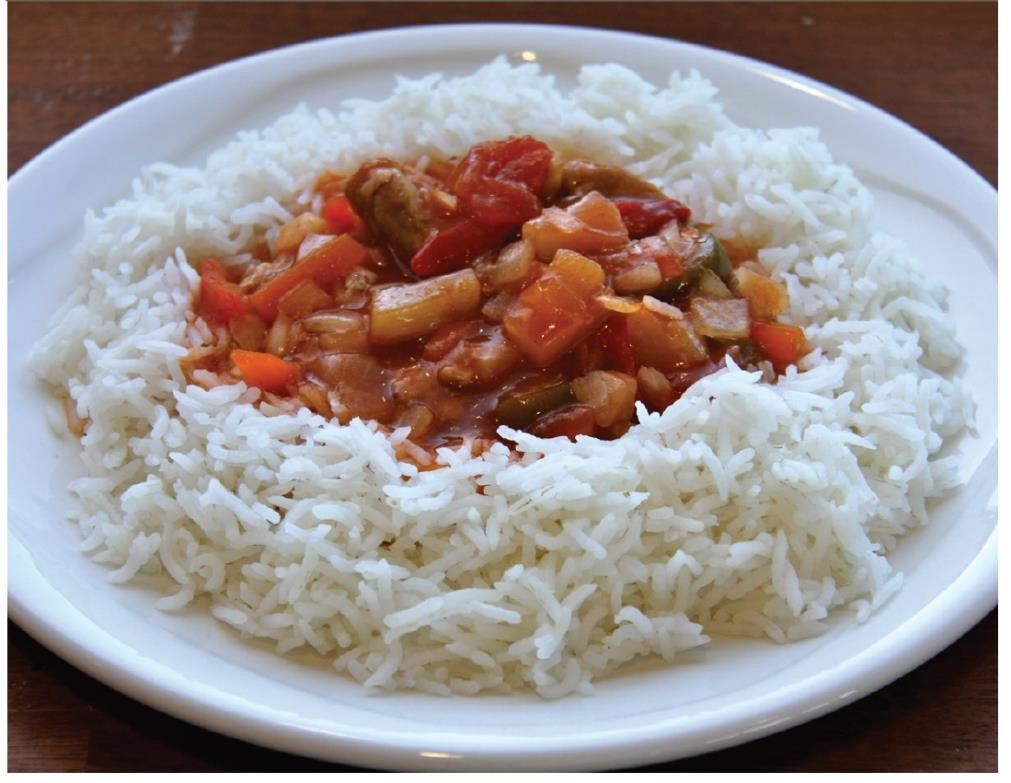 |
| Chicken and Leek Pie | 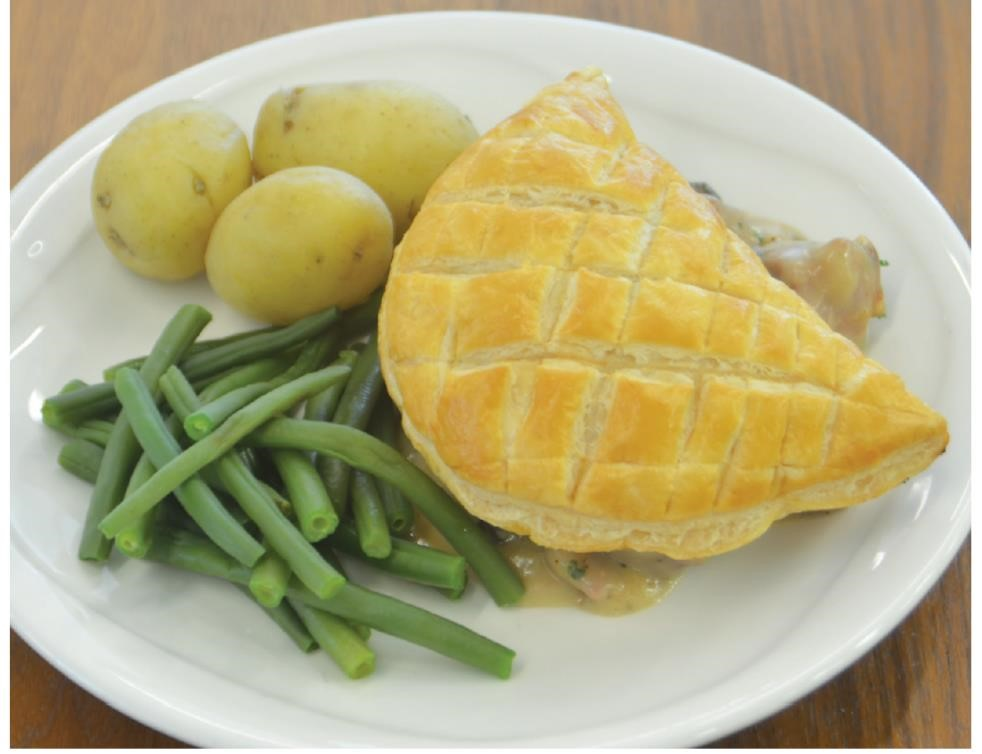 |
| Cumberland Sausages | 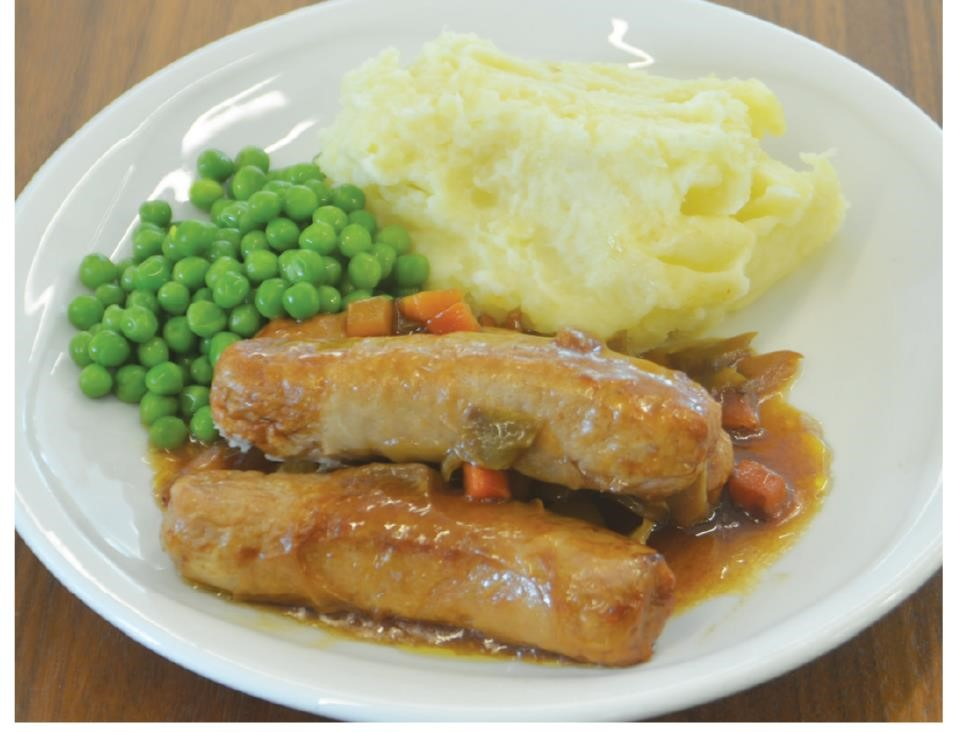 |
| Smoked Haddock Fishcakes | 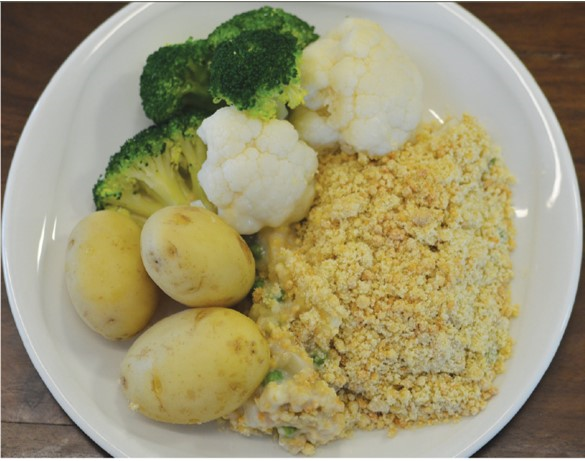 |

#### Distractor Task

Task 2 Introduction

Please complete the following tasks.

Q9

 How many houses are in this image?

________________________________________________________________

Q10 Which city is located in the United Kingdom (UK)?
Please select one option.

- London
- Kuala Lumpur
- Berlin

Q11

 How many children are in this image?

- 1 child
- 2 children
- 3 children

Q12
How many Street Lamps are in this image?

________________________________________________________________

Q13 From the following options, please tell us what your favourite mode of transport is.
Please select one option.

- Prefer not to say
- Car
- Train
- Coach
- Bicycle
- Other __________________________________________________

#### Baseline Survey 2

Q14 Please indicate your household income (total household income before tax)

- Less than £25K
- Between £25K and £39K
- £40K or above
- Prefer not to say

Q15 How often do you visit the planet Mars?

- Daily
- Once a week
- Once a month
- Once a year
- Never

|  |  |
| --- | --- |

Q16 On average, how many days a week do you eat meat for **lunch** (including fish, poultry, pork, beef and lamb)?

  For example, this could include a bacon, lettuce and tomato sandwich, or a tuna pasta salad.

- Never
- Less than once a week
- 1-2 days a week
- 3-4 days a week
- 5-6 days a week
- Every day of the week
- Prefer not to say

Q17 On average, how many days a week do you eat meat for **dinner** (including fish, poultry, pork, beef and lamb)?

 For example, this could include a shepherd’s pie, a beef lasagne, or fish and chips.

- Never
- Less than once a week
- 1-2 days a week
- 3-4 days a week
- 5-6 days a week
- Every day of the week
- Prefer not to say

Q18 How often do you use cafeterias and canteens to buy breakfast, lunch or dinner?

- Never
- Less than once a week
- 1-2 days a week
- 3-4 days a week
- 5-6 days a week
- Every day of the week
- Prefer not to say

### 1.2 Meal types available in the meal selection task

Table 1: Distribution of meals available across the five meal selection scenarios in the meal selection task, grouped according to meal type and environmental impact.

|  | *Environmental impact score of meal* | | |
| --- | --- | --- | --- |
| **Meal Type** | *Lowest* | *Medium* | *Highest* |
| Vegetarian | 3 | 1 | 0 |
| Fish containing | 0 | 0 | 1 |
| Poultry/Pork containing | 2 | 4 | 1 |
| Beef/lamb containing | 0 | 0 | 3 |

### 1.3 Model Variables

*Table 2: An explanation of variables used in the study’s analyses and which analyses the variables are used in.*

| **Variable (Values)** | **Explanation** | **Analyses** | **Type of Variable** |
| --- | --- | --- | --- |
| Participant.ID | Each participant has a unique ID | Manipulation Check  Primary Analyses | Categorical |
| Ecolabel Present (1 or 0) | Dummy variable explaining whether participant was assigned the control (no ecolabel) or an ecolabel treatment group. | Manipulation Check  Primary Analyses | Binary |
| Total Range (0-4) | Variable for the overall Range (the positive Range + negative Range). | Primary Analyses | Categorical (with levels) |
| Positive Range (0-2) | A measure of the lowest impact ecolabel available in each choice scenario in the meal selection task. If an A is available, score 2, if B, but not an A is available, score 1, and if C but not an A or B is available, score 0. | Secondary Analyses | Categorical (with levels) |
| Negative Range (0-2) | A measure of the highest impact ecolabel available in a given choice scenario in the meal selection task. If an E is available, score 2, if D, but not an E is available, score 1, and if C but not a D or E is available, score 0. |  | Categorical (with levels) |

### 1.4 Participant Characteristics

*Table 3: A explanation of the values that each participant characteristic can have, and the analyses each characteristic is included in.*

| **Participant Characteristic** | **Values/Explanation** | **Analyses** |
| --- | --- | --- |
| Age Group | - 18-34 - 35-49 - 50-64 - 65+ | Primary and exploratory Analyses |
| Gender | - Male - Female - Prefer to self identify | Primary and exploratory Analyses |
| Education Status | Highest levels of education received:   - Up to 4 or less GCSEs, or similar. - Up to 5GCSEs, A/AS Level or Apprenticeships, or similar. - Up to University Degree, or similar. | Primary and exploratory Analyses |
| Household Income | - Less than £25K - Between £25K and £39K - £40K or above - Prefer not to say. | Primary and exploratory Analyses |
| Device Type | - Mobile - Other | Primary and exploratory Analyses |
| Meat Consumption Frequency | Aggregate score (from 0 to 10) of lunch and dinner meat eating frequencies: whereby answers for both lunch and dinner questions were scored ‘Never’ (0), ‘Less than once a week’ (1), ‘1-2 days a week’ (2), ‘3-4 days a week’ (3), ‘5-6 days a week’ (4), ‘Every day of the week’ (5) | Exploratory analyses |
| Cafeteria attendance | Frequency of Canteen Going   - - Never   - Less than once a week   - 1-2 days a week   - 3-4 days a week   - 5-6 days a week   - Every day of the week | Exploratory analyses |

### 1.5 Deviations from protocol

In the secondary analysis, we did not include the variable “total ecolabel Range” in our models as this would have confounded with the variables positive and negative ecolabel.

In addition, the participant characteristic ‘Meat eating frequency’ for lunch and dinner was combined into one variable using a scoring system (0-10, whereby participants scored 0 if they don’t eat meat for lunch or dinner ever, and they scored 10 if they consumed meat every day for lunch and dinner.

The answers available for questions about meat consumption and frequency of canteen visits were different between the protocol vs the actual study (Table 4).

*Table 4: A comparison of the available choices that participants could select from for questions 16,17*

| **Relevant Question Number** | **Choices available in protocol** | **Choices available in the study survey** |
| --- | --- | --- |
| 16,17 | Meat Eating Frequency   - Less than once a week - 1 - 2 days a week - 3 - 5 days a week - 6 - 7 days a week | Meat Eating Frequency   - Never - Less than once a week - 1-2 days a week - 3-4 days a week - 5-6 days a week - Every day of the week |
| 18 | Frequency of Canteen Going   - Never - Less than once a week - 1 - 2 days a week - 3 - 4 days a week - 5 or more days a week | Frequency of Canteen Going   - Never - Less than once a week - 1-2 days a week - 3-4 days a week - 5-6 days a week - Every day of the week |

#### Primary Outcome Measure: explanation for deviation from protocol

We could not use the planned primary outcome measure as we believe that participants did not correctly complete the ranking task.

The primary outcome measure in the protocol was a binary outcome variable which scored each meal choice selection as 1 or 0. In the meal selection task, if a participant chose a more sustainable meal that they preferred less than another (less sustainable) meal option present (according to their indicated preferences from the ranking task), the meal selection was scored 1. If participants chose their preferred meal option (whether more or less sustainable), or a less preferred and less sustainable option, the meal selection scored 0.

Thus, the primary outcome variable relied on participants correctly ranking meals according to their preferences (1 = highest preference, 15 = lowest preference) in the meal ranking task. However, results from the ranking and meal selection task suggest that the ranking task was not completed correctly, and thus was not a good indicator of participants’ meal preferences.

Firstly, Figure 1 highlights the discrepancy between the preferences of participants for either the low, medium or high impact meal indicated in the ranking task, and the actual meal selected when the low, medium or high impact meal were presented side-by-side in the meal selection task. If participants were ranking meals according to their true preference, then we would expect that the ranking task and meal selection task bars in the control group would be roughly similar.

In the preference ranking task, all participants were initially presented with 15 meal choices in the same order (by meal type and from low, medium to high impact). Therefore, if participants only made minimal changes when reordering meal options, their final preference ranking would be very similar to the original ranking. This could explain the particularly large gap between low impact selections in Ranking task compared to Meal selection task (Figure 1).


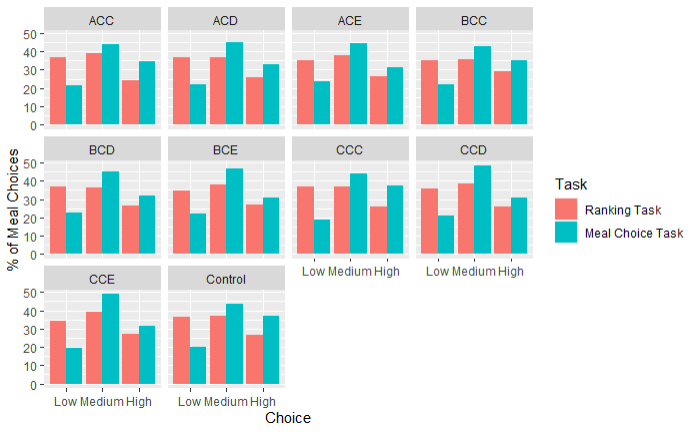


Figure 1: Bar charts to show the revealed preference of meals in ranking task and the meals chosen in meal selection task, grouped by the condition participants were assigned.

Secondly, 26% of all meal selections in the meal selection task were of meals that participants indicated were of lower preference and were less sustainable (Table 5), whilst only 10.5% of participants selected a meal that was more sustainable and lower preference than they indicated in the ranking task. We would expect the latter percentage to be larger than the former due to the expected effect of ecolabels encouraging participants to choose more sustainable options. Although we could hypothesise that the presence of ecolabels resulted in a backlash effect and encouraged participants to pick meals they liked less, and which were less sustainable – we see similar patterns when just looking at the control group with no ecolabels present (whereby 8.2% selected a more sustainable and less preferred meal and 26.5% selected a less sustainable and less preferred meal). This further suggests that participants did not complete the ranking task correctly.

Table 5: Quadrant of possible swaps made in the meal selection task from the indicated preference in the ranking task. The top right quadrant would score 1 in our protocol main outcome measure, all other quadrants score 0. MCT: Meal selection task and RT: Ranking task.

|  | **Less sustainable option picked in the Meal selection task (MCT)** | **More sustainable option picked in the Meal selection task (MCT)** |
| --- | --- | --- |
| **A swap was made:** the indicated preference did not match the meal selected in the meal selection task. | 26% of meal selections were an unsustainable swap.  (RT:L & MCT: M\|H or RT:M & MCT:H) | 10.5% of meal selections were sustainable swaps.  (RT:M & MCT:L or RT:H & MCT:L\|M) |
| **A swap wasn’t made:** the indicated preference matched the meal selected in the meal selection task. | 48.24% of meal selections matched the participant’s indicated preference, which was not the sustainable most option.  (RT:M & MCT:M or RT:H & MCT:H) | 15.21% of meal selections matched the participant’s indicated preference, which was the sustainable most option.  (RT:L&MCT:L) |

In addition, we analysed the degree to which participants re-ordered meals from the original ordering in the meal ranking task. Across 2603 participants, the median Spearman’s correlational value between the original rank and the final ranking order of meals was 0.48571. Of those participants, 1249 had a significant positive correlation at p<0.05 between the original ranking of meals, and their final preference ranking of meals. This suggests that participants may have left many of the meals in the original order, and thus may not have ranked each meal according to their true preference.

Finally, the setup of the preference ranking task was high effort and non-naturalistic in comparison to the meal selection task. The ranking task involved comparison of 15 diverse meal options, which is not representative of a day-to-day decision.

Taken together, this evidence suggests that the final ranking of meals by participants were not truly reflective of their actual meal preferences. These conclusions led to several deviations of the analysis from protocol. Firstly, we could not use the planned primary outcome measure, and in place created two new outcome measures (*Low* and *Low/Medium*). Additionally, we could not test whether meal preferences moderated the effects of ecolabels and their changing ranges on sustainable meal selection (secondary and sensitivity analyses). Finally, we did not construct the rank difference model as this was based upon the ranking task being properly collected.

*It should be noted that we cannot establish from these data alone whether an individual participant’s rank matched their true preferences (someone might have barely changed the order but did so carefully and it reflected their true preferences) – but looking across the pattern as a whole does give us a strong indication that participants did not truly rank their preferences.*

## Results

### 2.1 Primary Analysis – full table with demographics

*Table 6: Full outputs from two generalised linear mixed effect models, showing odds ratios, 95% confidence intervals and p-values for two models testing the effect of ecolabel Total Ranges and participant characteristics on LHS) the selection of the Low/Medium impact meals and RHS) the selection of the Low impact meal.*

|  | | *Outcome Measure* | | | | | | | |  |
| --- | --- | --- | --- | --- | --- | --- | --- | --- | --- | --- |
|  | | *Low/Medium* | | | | | *Low* | | |  |
| *Predictors* | | *Odds Ratios* | *CI* | | *P* | | *Odds Ratios* | *CI* | *P* |  |
| (Intercept) | | 3.59 | | 2.98 – 4.33 | **<0.001** | | 0.24 | 0.19 – 0.31 | **<0.001** |  |
| Ecolabel Total Range [0] | | 0.77 | | 0.65 – 0.91 | **0.003** | | 0.71 | 0.58 – 0.88 | **0.001** |  |
| Ecolabel Total Range [1] | | 0.92 | | 0.79 – 1.06 | 0.244 | | 0.82 | 0.69 – 0.98 | **0.028** |  |
| Ecolabel Total Range [2] | | 0.94 | | 0.82 – 1.08 | 0.400 | | 0.84 | 0.71 – 1.00 | **0.045** |  |
| Ecolabel Total Range [3] | | 0.98 | | 0.85 – 1.14 | 0.828 | | 0.88 | 0.74 – 1.05 | 0.151 |  |
| Age [35-49] | | 0.74 | | 0.66 – 0.84 | **<0.001** | | 1.14 | 0.96 – 1.35 | 0.123 |  |
| Age [50-64] | | 0.68 | | 0.60 – 0.77 | **<0.001** | | 1.20 | 1.00 – 1.42 | **0.044** |  |
| Age [65+] | | 0.58 | | 0.50 – 0.66 | **<0.001** | | 1.17 | 0.97 – 1.42 | 0.101 |  |
| Gender [Male] | | 0.73 | | 0.67 – 0.79 | **<0.001** | | 0.55 | 0.48 – 0.62 | **<0.001** |  |
| Gender [Prefer to self-identify] | | 0.91 | | 0.36 – 2.31 | 0.838 | | 1.37 | 0.42 – 4.48 | 0.599 |  |
| Education Status [Apprenticeship] | | 0.89 | | 0.68 – 1.16 | 0.380 | | 1.38 | 0.92 – 2.08 | 0.117 |  |
| Education Status  [Level 2 qualifications] | | 0.98 | | 0.86 – 1.12 | 0.788 | | 1.12 | 0.92 – 1.37 | 0.254 |  |
| Education Status  [Level 3 qualifications] | | 1.08 | | 0.94 – 1.25 | 0.288 | | 1.41 | 1.14 – 1.74 | **0.001** |  |
| Education Status  [Level 4 qualifications] | | 1.20 | | 1.06 – 1.36 | **0.004** | | 1.73 | 1.44 – 2.08 | **<0.001** |  |
| Education Status  [No qualifications] | | 0.93 | | 0.76 – 1.15 | 0.529 | | 0.93 | 0.67 – 1.28 | 0.648 |  |
| Education Status  [Other] | | 1.19 | | 0.82 – 1.70 | 0.358 | | 1.66 | 1.01 – 2.72 | **0.044** |  |
| Household Income  [Between £25K and £39K] | | 0.90 | | 0.81 – 1.00 | 0.054 | | 0.86 | 0.75 – 1.00 | **0.047** |  |
| Household Income  [Less than £25K] | | 0.93 | | 0.83 – 1.04 | 0.226 | | 0.82 | 0.70 – 0.96 | **0.016** |  |
| Household Income  [Prefer not to say] | | 1.03 | | 0.83 – 1.28 | 0.771 | | 1.13 | 0.85 – 1.51 | 0.391 |  |
| Device  [non-mobile] | | 0.93 | | 0.84 – 1.02 | 0.130 | | 1.09 | 0.95 – 1.25 | 0.217 |  |
| **Random Effects** | | | | | | | | | | |
| σ^2^ | 3.29 | | | | | 3.29 | | | | |
| τ_00_ _ResponseId_ | 0.09 | | | | | 0.65 | | | | |
| ICC | 0.03 | | | | | 0.17 | | | | |
| N _ResponseId_ | 2298 | | | | | 2298 | | | | |
| Observations | 11490 | | | | | 11490 | | | | |
| Marginal R^2^/Conditional R^2^ | 0.031/0.058 | | | | | 0.038/0.197 | | | | |

#### Both model intercepts are set at: Ecolabel Total Range = 4, Age = 18-34 years, Gender = Female, Education Status = Level 1 Qualifications, Household Income = More than £40k, Device = Mobile. Significant p-values (p<0.025) are highlighted by bold text.

#### Predicted Probabilities

*Table 7: Predicted probabilities for LHS) the selection of the Low/Medium impact meals and RHS) the selection of the Low impact meal.*

|  | ***Outcome Measure*** | | | |
| --- | --- | --- | --- | --- |
|  | ***Low/Medium*** | | ***Low*** | |
| Ecolabel Range | Predicted Probability | 95% Confidence interval | Predicted Probability | 95% Confidence interval |
|  |  |  |  |  |
| 0 | 0.73 | 0.28 – 0.95 | 0.15 | 0.02-0.55 |
| 1 | 0.77 | 0.31 – 0.96 | 0.17 | 0.03-0.59 |
| 2 | 0.77 | 0.32 – 0.96 | 0.17 | 0.03-0.60 |
| 3 | 0.78 | 0.33 – 0.96 | 0.18 | 0.03-0.61 |
| 4 | 0.78 | 0.33 – 0.96 | 0.20 | 0.03-0.64 |

*Both model intercepts are set at: Ecolabel Total Range = 4, Age = 18-34 years, Gender = Female, Education Status = Level 1 Qualifications, Household Income = More than £40k, Device = Mobile. Significant p-values (p<0.025) are highlighted by bold text.*

### 2.2 Primary Analysis – full table with demographics and intercept Range = 0

*Table 8: Full outputs from two generalised linear mixed effect models, showing odds ratios, 95% confidence intervals and p-values for two models testing the effect of ecolabel Total Ranges and participant characteristics on LHS) the selection of the Low/Medium impact meals and RHS) the selection of the Low impact meal.*

|  | | *Outcome Measure* | | | | | | |  |
| --- | --- | --- | --- | --- | --- | --- | --- | --- | --- |
|  | | *Low/Medium* | | | *Low* | | | |  |
| *Predictors* | | *Odds Ratios* | *CI* | *P* | *Odds Ratios* | | *CI* | *P* |  |
| (Intercept) | | 2.77 | 2.30 – 3.34 | **<0.001** | 0.17 | | 0.13 – 0.22 | **<0.001** |  |
| Ecolabel Total Range [1] | | 1.19 | 1.03 – 1.37 | **0.020** | 1.15 | | 0.96 – 1.39 | 0.130 |  |
| Ecolabel Total Range [2] | | 1.22 | 1.06 – 1.40 | **0.004** | 1.19 | | 1.00 – 1.41 | 0.056 |  |
| Ecolabel Total Range [3] | | 1.27 | 1.10 – 1.47 | **0.001** | 1.24 | | 1.03 – 1.49 | **0.025** |  |
| Ecolabel Total Range [4] | | 1.30 | 1.09 – 1.53 | **0.003** | 1.41 | | 1.14 – 1.74 | **0.001** |  |
| Age [35-49] | | 0.74 | 0.66 – 0.84 | **<0.001** | 1.14 | | 0.96 – 1.35 | 0.123 |  |
| Age [50-64] | | 0.68 | 0.60 – 0.77 | **<0.001** | 1.20 | | 1.00 – 1.42 | **0.044** |  |
| Age [65+] | | 0.58 | 0.50 – 0.66 | **<0.001** | 1.17 | | 0.97 – 1.42 | 0.101 |  |
| Gender [Male] | | 0.73 | 0.67 – 0.79 | **<0.001** | 0.55 | | 0.48 – 0.62 | **<0.001** |  |
| Gender [Prefer to self-identify] | | 0.91 | 0.36 – 2.31 | 0.838 | 1.37 | | 0.42 – 4.48 | 0.599 |  |
| Education Status [Apprenticeship] | | 0.89 | 0.68 – 1.16 | 0.380 | 1.38 | | 0.92 – 2.08 | 0.117 |  |
| Education Status  [Level 2 qualifications] | | 0.98 | 0.86 – 1.12 | 0.788 | 1.12 | | 0.92 – 1.37 | 0.254 |  |
| Education Status  [Level 3 qualifications] | | 1.08 | 0.94 – 1.25 | 0.288 | 1.41 | | 1.14 – 1.74 | **0.001** |  |
| Education Status  [Level 4 qualifications] | | 1.20 | 1.06 – 1.36 | **0.004** | 1.73 | | 1.44 – 2.08 | **<0.001** |  |
| Education Status  [No qualifications] | | 0.93 | 0.76 – 1.15 | 0.529 | 0.93 | | 0.67 – 1.28 | 0.648 |  |
| Education Status  [Other] | | 1.19 | 0.82 – 1.70 | 0.358 | 1.66 | | 1.01 – 2.72 | **0.044** |  |
| Household Income  [Between £25K and £39K] | | 0.90 | 0.81 – 1.00 | 0.054 | 0.86 | | 0.75 – 1.00 | **0.047** |  |
| Household Income  [Less than £25K] | | 0.93 | 0.83 – 1.04 | 0.226 | 0.82 | | 0.70 – 0.96 | **0.016** |  |
| Household Income  [Prefer not to say] | | 1.03 | 0.83 – 1.28 | 0.771 | 1.13 | | 0.85 – 1.51 | 0.391 |  |
| Device  [non-mobile] | | 0.93 | 0.84 – 1.02 | 0.130 | 1.09 | | 0.95 – 1.25 | 0.217 |  |
| **Random Effects** | | | | | | | | | |
| σ^2^ | 3.29 | | | | | 3.29 | | | |
| τ_00_ _ResponseId_ | 0.09 | | | | | 0.65 | | | |
| ICC | 0.03 | | | | | 0.17 | | | |
| N _ResponseId_ | 2298 | | | | | 2298 | | | |
| Observations | 11490 | | | | | 11490 | | | |
| Marginal R^2^/Conditional R^2^ | 0.031/0.058 | | | | | 0.038/0.197 | | | |

*Both model intercepts are set at: Ecolabel Total Range = 0, Age = 18-34 years, Gender = Female, Education Status = Level 1 Qualifications, Household Income = More than £40k, Device = Mobile. Significant p-values (p<0.025) are highlighted by bold text.*

### 2.3 Secondary Analysis

Total Range can be subdivided into Positive Range and Negative Range:

- Positive Range was a measure of how different the lowest impact ecolabel available in each choice scenario was from ‘C’. For example, if an ‘A’ was available, a score of 2 was given; if ‘B’, a score of 1; if ‘C’, it scored 0.
- Negative Range was similarly a measure of how different the highest impact ecolabel available in each choice scenario was from ‘C’ (see list below).

| **Ecolabels** | **Positive Range** | **Negative Range** | **Total Range** |
| --- | --- | --- | --- |
| ACE | 2 | 2 | 4 |
| ACD | 2 | 1 | 3 |
| BCE | 1 | 2 | 3 |
| ACC | 2 | 0 | 2 |
| BCD | 1 | 1 | 2 |
| CCE | 0 | 2 | 2 |
| BCC | 1 | 0 | 1 |
| CCD | 0 | 1 | 1 |
| CCC | 0 | 0 | 0 |

Participants that were presented with ecolabels that had a negative Range of 0 (ACC, BCC or CCC) had significantly lower odds of choosing Low/Medium impact meals compared to those presented with a negative Range of 2 (ACE) (Low/Medium: OR: 0.81, CI: 0.74-0.89, p<0.001). There were no significant differences in the odds of choosing the Low/Medium impact meals between other positive and negative Ranges (Table 9).

Participants that were presented with ecolabels that had a positive Range of 0 (CCC, CCD, or CCE) had significantly lower odds of choosing the Low impact meal compared to those presented with a positive Range of 2 (ACE) (Low: OR: 0.83, CI:0.73-0.93, p=0.002). There were no significant differences in the odds of choosing the Low impact meal between other positive and negative Ranges (Table 9).

Table 9: Outputs from two generalised linear mixed effect models, showing odds ratios, 95% confidence intervals and p-values for two models testing the effect of ecolabel positive and negative Ranges and participant characteristics on LHS) selection of Low/Medium impact meals and RHS) on the selection of the Low impact meal.

|  | Outcome Measure | | | | | |
| --- | --- | --- | --- | --- | --- | --- |
|  | *Low/Medium* | | | *Low* | | |
|  | *Odds Ratios* | *CI* | *p* | *Odds Ratios* | *CI* | *P* |
| Positive Range [0] | 0.99 | 0.90 – 1.09 | 0.807 | 0.83 | 0.73 – 0.93 | **0.002** |
| Positive Range [1] | 1.01 | 0.91 – 1.11 | 0.857 | 0.97 | 0.86 – 1.09 | 0.588 |
| Negative Range [0] | 0.81 | 0.74 – 0.89 | **<0.001** | 0.92 | 0.81 – 1.04 | 0.170 |
| Negative Range [1] | 0.97 | 0.88 – 1.07 | 0.555 | 1.00 | 0.89 – 1.13 | 0.967 |

*Both model intercepts are set at: Ecolabel Total Range = 4, Age = 18-34 years, Gender = Female, Education Status = Level 1 Qualifications, Household Income = More than £40k, Device = Mobile. Significant p-values (p<0.005) are highlighted by bold text.*

### 2.4 Effects of Participant Characteristics on meal selection

Male participants had significantly lower odds of selecting a Low/Medium or the Low impact meal than female participants (Low/Medium: OR: 0.73, CI:0.67-0.79, Low: OR: 0.55, CI:0.48-0.62). Those aged above 35 years had significantly lower odds of selecting a Low/Medium impact meal than those aged below 35 years (Low/Medium: Age[35-49]: OR: 0.74, CI: 0.66-0.84; Age [50-64]: OR: 0.68, CI: 0.60 – 0.77; Age [65+]: OR: 0.58, CI: 0.50 – 0.66**)** but there was no significant effect of age on selection of Low impact meals. Participants that achieved a Level 3 or 4 qualification had significantly higher odds of selecting a Low impact meal in comparison to those that had achieved a Level 1 qualification (Low: Education Status [Level 3 Qualifications] OR: 1.41, CI:1.14-1.74; Education Status [Level 4 Qualifications] OR: 1.73, CI: 1.44 – 2.08). Only those that achieved a Level 4 qualification had significantly higher odds of selecting a Low/Medium impact meal in comparison to those that had achieved a Level 1 qualification (Low/Medium: Education Status [Level 4 Qualifications] OR: 1.20, CI: 1.06-1.3). Participants that had a household income of less than £25k had lower odds of choosing the Low impact meal than those earning above £40k (Low: Household Income [Less than £25K]: OR: 0.82, CI:0.70-0.96). There were no other significant effects of other participant characteristics on either outcome measure.

### 2.5 Models Including Interactions between Participant Characteristics and Ecolabel range

*Model intercepts are set at: Ecolabel Total Range = 4, Age = 18-34 years, Gender = Female, Education Status = Level 1 Qualifications, Household Income = More than £40k, Device = Mobile. Significant p-values (p<0.025) are highlighted by bold text.*

#### Gender

*Table 10a: Outcome measure: Low/medium*

|  |  | | |
| --- | --- | --- | --- |
| *Predictors* | *Odds Ratios* | *CI* | *p* |
| (Intercept) | 3.78 | 3.02 – 4.73 | **<0.001** |
| Ecolabel Total Range [0] | 0.68 | 0.54 – 0.87 | **0.002** |
| Ecolabel Total Range [1] | 0.87 | 0.70 – 1.07 | 0.184 |
| Ecolabel Total Range [2] | 0.90 | 0.73 – 1.10 | 0.291 |
| Ecolabel Total Range [3] | 0.94 | 0.76 – 1.17 | 0.584 |
| Education Status [Apprenticeship] | 0.89 | 0.68 – 1.16 | 0.386 |
| Education Status [Level 2 qualifications 5 or more GCSEs (A* to C or 9 to 4), O levels (passes), CSEs (grade 1), School Certification, 1 A level, 2 to 3 AS levels, VCEs, Intermediate or Higher Diploma, Welsh Baccalaureate Intermediate Diploma, NVQ level 2, Intermediate GNVQ, City and Guilds Craft, BTEC First or General Diploma, RSA Diploma] | 0.99 | 0.86 – 1.13 | 0.846 |
| Education Status [Level 3 qualifications 2 or more A levels or VCEs, 4 or more AS levels, Higher School Certificate, Progression or Advanced Diploma, Welsh Baccalaureate Advance Diploma, NVQ level 3; Advanced GNVQ, City and Guilds Advanced Craft, ONC, OND, BTEC National, RSA Advanced Diploma] | 1.08 | 0.93 – 1.25 | 0.308 |
| Education Status [Level 4 qualifications or above  degree (BA, BSc), higher degree (MA, PhD, PGCE), NVQ level 4 to 5, HNC, HND, RSA Higher Diploma, BTEC Higher level, professional qualifications (for example, teaching, nursing, accountancy)] | 1.20 | 1.06 – 1.37 | **0.004** |
| Education Status [No qualifications] | 0.93 | 0.76 – 1.15 | 0.528 |
| Education Status [Other  vocational or work-related qualifications, other qualifications achieved in England or Wales, qualifications achieved outside England or Wales (equivalent not stated or unknown)] | 1.18 | 0.82 – 1.70 | 0.361 |
| Household Income [Between £25K and £39K] | 0.90 | 0.81 – 1.00 | 0.051 |
| Household Income [Less than £25K] | 0.93 | 0.83 – 1.04 | 0.228 |
| Household Income [Prefer not to say] | 1.05 | 0.85 – 1.30 | 0.641 |
| Device [non-mobile] | 0.93 | 0.84 – 1.02 | 0.124 |
| Age [35-49] | 0.74 | 0.66 – 0.84 | **<0.001** |
| Age [50-64] | 0.68 | 0.60 – 0.77 | **<0.001** |
| Age [65+] | 0.58 | 0.50 – 0.66 | **<0.001** |
| Gender [Male] | 0.66 | 0.51 – 0.84 | **0.001** |
| Ecolabel Total Range [0] x Gender [Male] | 1.27 | 0.90 – 1.78 | 0.168 |
| Ecolabel Total Range [1] x Gender [Male] | 1.11 | 0.82 – 1.49 | 0.492 |
| Ecolabel Total Range [2] x Gender [Male] | 1.11 | 0.84 – 1.47 | 0.475 |
| Ecolabel Total Range [3] x Gender [Male] | 1.09 | 0.80 – 1.46 | 0.592 |
| **Random Effects** | | | |
| σ^2^ | 3.29 | | |
| τ_00_ _ResponseId_ | 0.10 | | |
| ICC | 0.03 | | |
| N _ResponseId_ | 2293 | | |
| Observations | 11465 | | |
| Marginal R^2^ / Conditional R^2^ | 0.032 / 0.059 | | |

*Table 10b: Outcome measure: Low*

|  |  | | |
| --- | --- | --- | --- |
| *Predictors* | *Odds Ratios* | *CI* | *P* |
| (Intercept) | 0.25 | 0.19 – 0.33 | **<0.001** |
| Ecolabel Total Range [0] | 0.70 | 0.53 – 0.92 | **0.011** |
| Ecolabel Total Range [1] | 0.79 | 0.63 – 1.00 | 0.052 |
| Ecolabel Total Range [2] | 0.80 | 0.64 – 0.99 | **0.043** |
| Ecolabel Total Range [3] | 0.84 | 0.67 – 1.07 | 0.155 |
| Education Status [Apprenticeship] | 1.38 | 0.92 – 2.07 | 0.120 |
| Education Status [Level 2 qualifications 5 or more GCSEs (A* to C or 9 to 4), O levels (passes), CSEs (grade 1), School Certification, 1 A level, 2 to 3 AS levels, VCEs, Intermediate or Higher Diploma, Welsh Baccalaureate Intermediate Diploma, NVQ level 2, Intermediate GNVQ, City and Guilds Craft, BTEC First or General Diploma, RSA Diploma] | 1.12 | 0.92 – 1.37 | 0.247 |
| Education Status [Level 3 qualifications 2 or more A levels or VCEs, 4 or more AS levels, Higher School Certificate, Progression or Advanced Diploma, Welsh Baccalaureate Advance Diploma, NVQ level 3; Advanced GNVQ, City and Guilds Advanced Craft, ONC, OND, BTEC National, RSA Advanced Diploma] | 1.41 | 1.14 – 1.74 | **0.002** |
| Education Status [Level 4 qualifications or above  degree (BA, BSc), higher degree (MA, PhD, PGCE), NVQ level 4 to 5, HNC, HND, RSA Higher Diploma, BTEC Higher level, professional qualifications (for example, teaching, nursing, accountancy)] | 1.72 | 1.44 – 2.07 | **<0.001** |
| Education Status [No qualifications] | 0.93 | 0.67 – 1.29 | 0.673 |
| Education Status [Other  vocational or work-related qualifications, other qualifications achieved in England or Wales, qualifications achieved outside England or Wales (equivalent not stated or unknown)] | 1.65 | 1.01 – 2.71 | **0.046** |
| Household Income [Between £25K and £39K] | 0.86 | 0.74 – 1.00 | **0.043** |
| Household Income [Less than £25K] | 0.82 | 0.70 – 0.96 | **0.014** |
| Household Income [Prefer not to say] | 1.15 | 0.87 – 1.54 | 0.326 |
| Device [non-mobile] | 1.09 | 0.95 – 1.25 | 0.218 |
| Age [35-49] | 1.14 | 0.97 – 1.35 | 0.119 |
| Age [50-64] | 1.20 | 1.01 – 1.43 | **0.042** |
| Age [65+] | 1.18 | 0.97 – 1.42 | 0.096 |
| Gender [Male] | 0.50 | 0.37 – 0.68 | **<0.001** |
| Ecolabel Total Range [0] x Gender [Male] | 1.03 | 0.67 – 1.58 | 0.891 |
| Ecolabel Total Range [1] x Gender [Male] | 1.07 | 0.75 – 1.53 | 0.708 |
| Ecolabel Total Range [2] x Gender [Male] | 1.14 | 0.81 – 1.60 | 0.451 |
| Ecolabel Total Range [3] x Gender [Male] | 1.10 | 0.77 – 1.58 | 0.589 |
| **Random Effects** | | | |
| σ^2^ | 3.29 | | |
| τ_00_ _ResponseId_ | 0.66 | | |
| ICC | 0.17 | | |
| N _ResponseId_ | 2293 | | |
| Observations | 11465 | | |
| Marginal R^2^ / Conditional R^2^ | 0.038 / 0.198 | | |

#### Age

*Table 11a: Outcome measure: Low/medium*

|  |  | | |
| --- | --- | --- | --- |
| *Predictors* | *Odds Ratios* | *CI* | *p* |
| (Intercept) | 4.49 | 3.37 – 5.99 | **<0.001** |
| Ecolabel Total Range [0] | 0.58 | 0.41 – 0.81 | **0.002** |
| Ecolabel Total Range [1] | 0.79 | 0.58 – 1.08 | 0.136 |
| Ecolabel Total Range [2] | 0.69 | 0.51 – 0.92 | **0.012** |
| Ecolabel Total Range [3] | 0.79 | 0.57 – 1.08 | 0.132 |
| Gender [Male] | 0.73 | 0.67 – 0.79 | **<0.001** |
| Gender [Prefer to self-identify] | 0.93 | 0.36 – 2.36 | 0.871 |
| Education Status [Apprenticeship] | 0.88 | 0.67 – 1.16 | 0.370 |
| Education Status [Level 2 qualifications 5 or more GCSEs (A* to C or 9 to 4), O levels (passes), CSEs (grade 1), School Certification, 1 A level, 2 to 3 AS levels, VCEs, Intermediate or Higher Diploma, Welsh Baccalaureate Intermediate Diploma, NVQ level 2, Intermediate GNVQ, City and Guilds Craft, BTEC First or General Diploma, RSA Diploma] | 0.99 | 0.86 – 1.13 | 0.828 |
| Education Status [Level 3 qualifications 2 or more A levels or VCEs, 4 or more AS levels, Higher School Certificate, Progression or Advanced Diploma, Welsh Baccalaureate Advance Diploma, NVQ level 3; Advanced GNVQ, City and Guilds Advanced Craft, ONC, OND, BTEC National, RSA Advanced Diploma] | 1.09 | 0.94 – 1.26 | 0.273 |
| Education Status [Level 4 qualifications or above  degree (BA, BSc), higher degree (MA, PhD, PGCE), NVQ level 4 to 5, HNC, HND, RSA Higher Diploma, BTEC Higher level, professional qualifications (for example, teaching, nursing, accountancy)] | 1.20 | 1.06 – 1.36 | **0.004** |
| Education Status [No qualifications] | 0.93 | 0.76 – 1.15 | 0.524 |
| Education Status [Other  vocational or work-related qualifications, other qualifications achieved in England or Wales, qualifications achieved outside England or Wales (equivalent not stated or unknown)] | 1.18 | 0.82 – 1.70 | 0.374 |
| Household Income [Between £25K and £39K] | 0.90 | 0.81 – 1.00 | 0.050 |
| Household Income [Less than £25K] | 0.93 | 0.83 – 1.04 | 0.217 |
| Household Income [Prefer not to say] | 1.03 | 0.84 – 1.28 | 0.768 |
| Device [non-mobile] | 0.93 | 0.84 – 1.02 | 0.132 |
| Age [35-49] | 0.53 | 0.37 – 0.76 | **0.001** |
| Age [50-64] | 0.49 | 0.34 – 0.70 | **<0.001** |
| Age [65+] | 0.46 | 0.32 – 0.66 | **<0.001** |
| Ecolabel Total Range [0] x Age [35-49] | 1.31 | 0.81 – 2.12 | 0.275 |
| Ecolabel Total Range [1] x Age [35-49] | 1.21 | 0.79 – 1.86 | 0.373 |
| Ecolabel Total Range [2] x Age [35-49] | 1.65 | 1.10 – 2.47 | **0.016** |
| Ecolabel Total Range [3] x Age [35-49] | 1.52 | 0.98 – 2.34 | 0.059 |
| Ecolabel Total Range [0] x Age [50-64] | 1.59 | 0.98 – 2.58 | 0.061 |
| Ecolabel Total Range [1] x Age [50-64] | 1.27 | 0.83 – 1.95 | 0.278 |
| Ecolabel Total Range [2] x Age [50-64] | 1.62 | 1.08 – 2.43 | **0.020** |
| Ecolabel Total Range [3] x Age [50-64] | 1.31 | 0.85 – 2.01 | 0.223 |
| Ecolabel Total Range [0] x Age [65+] | 1.53 | 0.94 – 2.48 | 0.085 |
| Ecolabel Total Range [1] x Age [65+] | 1.16 | 0.76 – 1.79 | 0.486 |
| Ecolabel Total Range [2] x Age [65+] | 1.32 | 0.88 – 1.97 | 0.182 |
| Ecolabel Total Range [3] x Age [65+] | 1.23 | 0.80 – 1.89 | 0.344 |
| **Random Effects** | | | |
| σ^2^ | 3.29 | | |
| τ_00_ _ResponseId_ | 0.09 | | |
| ICC | 0.03 | | |
| N _ResponseId_ | 2298 | | |
| Observations | 11490 | | |
| Marginal R^2^ / Conditional R^2^ | 0.033 / 0.060 | | |

*Table 11b: Outcome measure: Low*

|  |  | | |
| --- | --- | --- | --- |
| *Predictors* | *Odds Ratios* | *CI* | *p* |
| (Intercept) | 0.24 | 0.17 – 0.33 | **<0.001** |
| Ecolabel Total Range [0] | 0.60 | 0.39 – 0.90 | **0.014** |
| Ecolabel Total Range [1] | 0.76 | 0.54 – 1.07 | 0.111 |
| Ecolabel Total Range [2] | 0.93 | 0.68 – 1.28 | 0.675 |
| Ecolabel Total Range [3] | 0.90 | 0.65 – 1.27 | 0.560 |
| Gender [Male] | 0.54 | 0.48 – 0.62 | **<0.001** |
| Gender [Prefer to self-identify] | 1.40 | 0.43 – 4.59 | 0.576 |
| Education Status [Apprenticeship] | 1.38 | 0.92 – 2.08 | 0.118 |
| Education Status [Level 2 qualifications 5 or more GCSEs (A* to C or 9 to 4), O levels (passes), CSEs (grade 1), School Certification, 1 A level, 2 to 3 AS levels, VCEs, Intermediate or Higher Diploma, Welsh Baccalaureate Intermediate Diploma, NVQ level 2, Intermediate GNVQ, City and Guilds Craft, BTEC First or General Diploma, RSA Diploma] | 1.12 | 0.92 – 1.37 | 0.261 |
| Education Status [Level 3 qualifications 2 or more A levels or VCEs, 4 or more AS levels, Higher School Certificate, Progression or Advanced Diploma, Welsh Baccalaureate Advance Diploma, NVQ level 3; Advanced GNVQ, City and Guilds Advanced Craft, ONC, OND, BTEC National, RSA Advanced Diploma] | 1.41 | 1.14 – 1.74 | **0.001** |
| Education Status [Level 4 qualifications or above  degree (BA, BSc), higher degree (MA, PhD, PGCE), NVQ level 4 to 5, HNC, HND, RSA Higher Diploma, BTEC Higher level, professional qualifications (for example, teaching, nursing, accountancy)] | 1.73 | 1.44 – 2.08 | **<0.001** |
| Education Status [No qualifications] | 0.92 | 0.67 – 1.28 | 0.624 |
| Education Status [Other  vocational or work-related qualifications, other qualifications achieved in England or Wales, qualifications achieved outside England or Wales (equivalent not stated or unknown)] | 1.65 | 1.01 – 2.70 | **0.046** |
| Household Income [Between £25K and £39K] | 0.86 | 0.75 – 1.00 | **0.049** |
| Household Income [Less than £25K] | 0.82 | 0.70 – 0.96 | **0.016** |
| Household Income [Prefer not to say] | 1.14 | 0.86 – 1.51 | 0.376 |
| Device [non-mobile] | 1.09 | 0.95 – 1.25 | 0.202 |
| Age [35-49] | 1.34 | 0.90 – 1.99 | 0.153 |
| Age [50-64] | 0.99 | 0.65 – 1.51 | 0.968 |
| Age [65+] | 1.22 | 0.80 – 1.87 | 0.357 |
| Ecolabel Total Range [0] x Age [35-49] | 0.91 | 0.51 – 1.64 | 0.756 |
| Ecolabel Total Range [1] x Age [35-49] | 0.99 | 0.61 – 1.61 | 0.978 |
| Ecolabel Total Range [2] x Age [35-49] | 0.74 | 0.47 – 1.17 | 0.197 |
| Ecolabel Total Range [3] x Age [35-49] | 0.81 | 0.50 – 1.31 | 0.384 |
| Ecolabel Total Range [0] x Age [50-64] | 1.58 | 0.87 – 2.85 | 0.134 |
| Ecolabel Total Range [1] x Age [50-64] | 1.34 | 0.81 – 2.21 | 0.250 |
| Ecolabel Total Range [2] x Age [50-64] | 1.09 | 0.68 – 1.74 | 0.726 |
| Ecolabel Total Range [3] x Age [50-64] | 1.24 | 0.75 – 2.03 | 0.402 |
| Ecolabel Total Range [0] x Age [65+] | 1.46 | 0.80 – 2.65 | 0.219 |
| Ecolabel Total Range [1] x Age [65+] | 1.05 | 0.64 – 1.74 | 0.843 |
| Ecolabel Total Range [2] x Age [65+] | 0.82 | 0.51 – 1.32 | 0.416 |
| Ecolabel Total Range [3] x Age [65+] | 0.90 | 0.54 – 1.49 | 0.677 |
| **Random Effects** | | | |
| σ^2^ | 3.29 | | |
| τ_00_ _ResponseId_ | 0.66 | | |
| ICC | 0.17 | | |
| N _ResponseId_ | 2298 | | |
| Observations | 11490 | | |
| Marginal R^2^ / Conditional R^2^ | 0.039 / 0.200 | | |

#### Highest educational qualification

*Table 12a: Outcome measure: Low/medium*

|  |  | | |
| --- | --- | --- | --- |
| *Predictors* | *Odds Ratios* | *CI* | *p* |
| (Intercept) | 3.32 | 2.45 – 4.51 | **<0.001** |
| Ecolabel Total Range [0] | 1.00 | 0.67 – 1.49 | 1.000 |
| Ecolabel Total Range [1] | 0.98 | 0.69 – 1.39 | 0.897 |
| Ecolabel Total Range [2] | 1.04 | 0.75 – 1.44 | 0.833 |
| Ecolabel Total Range [3] | 1.00 | 0.71 – 1.41 | 0.995 |
| Gender [Male] | 0.73 | 0.67 – 0.79 | **<0.001** |
| Gender [Prefer to self-identify] | 0.93 | 0.36 – 2.37 | 0.875 |
| Household Income [Between £25K and £39K] | 0.90 | 0.81 – 1.00 | 0.055 |
| Household Income [Less than £25K] | 0.93 | 0.83 – 1.04 | 0.226 |
| Household Income [Prefer not to say] | 1.03 | 0.83 – 1.27 | 0.793 |
| Device [non-mobile] | 0.93 | 0.84 – 1.02 | 0.130 |
| Age [35-49] | 0.74 | 0.65 – 0.83 | **<0.001** |
| Age [50-64] | 0.68 | 0.60 – 0.77 | **<0.001** |
| Age [65+] | 0.58 | 0.51 – 0.66 | **<0.001** |
| Education Status [Apprenticeship] | 1.05 | 0.49 – 2.24 | 0.892 |
| Education Status [Level 2 qualifications 5 or more GCSEs (A* to C or 9 to 4), O levels (passes), CSEs (grade 1), School Certification, 1 A level, 2 to 3 AS levels, VCEs, Intermediate or Higher Diploma, Welsh Baccalaureate Intermediate Diploma, NVQ level 2, Intermediate GNVQ, City and Guilds Craft, BTEC First or General Diploma, RSA Diploma] | 1.06 | 0.72 – 1.57 | 0.755 |
| Education Status [Level 3 qualifications 2 or more A levels or VCEs, 4 or more AS levels, Higher School Certificate, Progression or Advanced Diploma, Welsh Baccalaureate Advance Diploma, NVQ level 3; Advanced GNVQ, City and Guilds Advanced Craft, ONC, OND, BTEC National, RSA Advanced Diploma] | 1.44 | 0.93 – 2.24 | 0.104 |
| Education Status [Level 4 qualifications or above  degree (BA, BSc), higher degree (MA, PhD, PGCE), NVQ level 4 to 5, HNC, HND, RSA Higher Diploma, BTEC Higher level, professional qualifications (for example, teaching, nursing, accountancy)] | 1.21 | 0.85 – 1.72 | 0.280 |
| Education Status [No qualifications] | 1.06 | 0.60 – 1.87 | 0.841 |
| Education Status [Other  vocational or work-related qualifications, other qualifications achieved in England or Wales, qualifications achieved outside England or Wales (equivalent not stated or unknown)] | 1.75 | 0.61 – 5.07 | 0.300 |
| Ecolabel Total Range [0] x Education Status [Apprenticeship] | 0.44 | 0.15 – 1.30 | 0.139 |
| Ecolabel Total Range [1] x Education Status [Apprenticeship] | 0.85 | 0.34 – 2.14 | 0.725 |
| Ecolabel Total Range [2] x Education Status [Apprenticeship] | 0.73 | 0.30 – 1.75 | 0.481 |
| Ecolabel Total Range [3] x Education Status [Apprenticeship] | 1.31 | 0.51 – 3.38 | 0.571 |
| Ecolabel Total Range [0] x Education Status [Level 2 qualifications 5 or more GCSEs (A* to C or 9 to 4), O levels (passes), CSEs (grade 1), School Certification, 1 A level, 2 to 3 AS levels, VCEs, Intermediate or Higher Diploma, Welsh Baccalaureate Intermediate Diploma, NVQ level 2, Intermediate GNVQ, City and Guilds Craft, BTEC First or General Diploma, RSA Diploma] | 0.83 | 0.48 – 1.43 | 0.503 |
| Ecolabel Total Range [1] x Education Status [Level 2 qualifications 5 or more GCSEs (A* to C or 9 to 4), O levels (passes), CSEs (grade 1), School Certification, 1 A level, 2 to 3 AS levels, VCEs, Intermediate or Higher Diploma, Welsh Baccalaureate Intermediate Diploma, NVQ level 2, Intermediate GNVQ, City and Guilds Craft, BTEC First or General Diploma, RSA Diploma] | 0.92 | 0.57 – 1.48 | 0.725 |
| Ecolabel Total Range [2] x Education Status [Level 2 qualifications 5 or more GCSEs (A* to C or 9 to 4), O levels (passes), CSEs (grade 1), School Certification, 1 A level, 2 to 3 AS levels, VCEs, Intermediate or Higher Diploma, Welsh Baccalaureate Intermediate Diploma, NVQ level 2, Intermediate GNVQ, City and Guilds Craft, BTEC First or General Diploma, RSA Diploma] | 0.87 | 0.55 – 1.35 | 0.528 |
| Ecolabel Total Range [3] x Education Status [Level 2 qualifications 5 or more GCSEs (A* to C or 9 to 4), O levels (passes), CSEs (grade 1), School Certification, 1 A level, 2 to 3 AS levels, VCEs, Intermediate or Higher Diploma, Welsh Baccalaureate Intermediate Diploma, NVQ level 2, Intermediate GNVQ, City and Guilds Craft, BTEC First or General Diploma, RSA Diploma] | 1.04 | 0.64 – 1.66 | 0.886 |
| Ecolabel Total Range [0] x Education Status [Level 3 qualifications 2 or more A levels or VCEs, 4 or more AS levels, Higher School Certificate, Progression or Advanced Diploma, Welsh Baccalaureate Advance Diploma, NVQ level 3; Advanced GNVQ, City and Guilds Advanced Craft, ONC, OND, BTEC National, RSA Advanced Diploma] | 0.57 | 0.32 – 1.04 | 0.066 |
| Ecolabel Total Range [1] x Education Status [Level 3 qualifications 2 or more A levels or VCEs, 4 or more AS levels, Higher School Certificate, Progression or Advanced Diploma, Welsh Baccalaureate Advance Diploma, NVQ level 3; Advanced GNVQ, City and Guilds Advanced Craft, ONC, OND, BTEC National, RSA Advanced Diploma] | 0.88 | 0.52 – 1.50 | 0.634 |
| Ecolabel Total Range [2] x Education Status [Level 3 qualifications 2 or more A levels or VCEs, 4 or more AS levels, Higher School Certificate, Progression or Advanced Diploma, Welsh Baccalaureate Advance Diploma, NVQ level 3; Advanced GNVQ, City and Guilds Advanced Craft, ONC, OND, BTEC National, RSA Advanced Diploma] | 0.75 | 0.45 – 1.23 | 0.251 |
| Ecolabel Total Range [3] x Education Status [Level 3 qualifications 2 or more A levels or VCEs, 4 or more AS levels, Higher School Certificate, Progression or Advanced Diploma, Welsh Baccalaureate Advance Diploma, NVQ level 3; Advanced GNVQ, City and Guilds Advanced Craft, ONC, OND, BTEC National, RSA Advanced Diploma] | 0.65 | 0.39 – 1.10 | 0.112 |
| Ecolabel Total Range [0] x Education Status [Level 4 qualifications or above  degree (BA, BSc), higher degree (MA, PhD, PGCE), NVQ level 4 to 5, HNC, HND, RSA Higher Diploma, BTEC Higher level, professional qualifications (for example, teaching, nursing, accountancy)] | 0.77 | 0.47 – 1.26 | 0.297 |
| Ecolabel Total Range [1] x Education Status [Level 4 qualifications or above  degree (BA, BSc), higher degree (MA, PhD, PGCE), NVQ level 4 to 5, HNC, HND, RSA Higher Diploma, BTEC Higher level, professional qualifications (for example, teaching, nursing, accountancy)] | 0.97 | 0.63 – 1.48 | 0.875 |
| Ecolabel Total Range [2] x Education Status [Level 4 qualifications or above  degree (BA, BSc), higher degree (MA, PhD, PGCE), NVQ level 4 to 5, HNC, HND, RSA Higher Diploma, BTEC Higher level, professional qualifications (for example, teaching, nursing, accountancy)] | 1.02 | 0.68 – 1.52 | 0.935 |
| Ecolabel Total Range [3] x Education Status [Level 4 qualifications or above  degree (BA, BSc), higher degree (MA, PhD, PGCE), NVQ level 4 to 5, HNC, HND, RSA Higher Diploma, BTEC Higher level, professional qualifications (for example, teaching, nursing, accountancy)] | 1.11 | 0.73 – 1.69 | 0.635 |
| Ecolabel Total Range [0] x Education Status [No qualifications] | 0.86 | 0.38 – 1.99 | 0.729 |
| Ecolabel Total Range [1] x Education Status [No qualifications] | 0.98 | 0.48 – 2.01 | 0.962 |
| Ecolabel Total Range [2] x Education Status [No qualifications] | 0.76 | 0.39 – 1.48 | 0.413 |
| Ecolabel Total Range [3] x Education Status [No qualifications] | 0.93 | 0.47 – 1.85 | 0.836 |
| Ecolabel Total Range [0] x Education Status [Other vocational or work-related qualifications, other qualifications achieved in England or Wales, qualifications achieved outside England or Wales (equivalent not stated or unknown)] | 0.48 | 0.12 – 1.93 | 0.299 |
| Ecolabel Total Range [1] x Education Status [Other vocational or work-related qualifications, other qualifications achieved in England or Wales, qualifications achieved outside England or Wales (equivalent not stated or unknown)] | 0.47 | 0.13 – 1.65 | 0.239 |
| Ecolabel Total Range [2] x Education Status [Other vocational or work-related qualifications, other qualifications achieved in England or Wales, qualifications achieved outside England or Wales (equivalent not stated or unknown)] | 0.70 | 0.21 – 2.35 | 0.562 |
| Ecolabel Total Range [3] x Education Status [Other vocational or work-related qualifications, other qualifications achieved in England or Wales, qualifications achieved outside England or Wales (equivalent not stated or unknown)] | 1.23 | 0.28 – 5.37 | 0.783 |
| **Random Effects** | | | |
| σ^2^ | 3.29 | | |
| τ_00_ _ResponseId_ | 0.09 | | |
| ICC | 0.03 | | |
| N _ResponseId_ | 2298 | | |
| Observations | 11490 | | |
| Marginal R^2^ / Conditional R^2^ | 0.034 / 0.060 | | |

*Table 12b: Outcome measure: Low*

|  |  | | |
| --- | --- | --- | --- |
| *Predictors* | *Odds Ratios* | *CI* | *p* |
| (Intercept) | 0.17 | 0.11 – 0.27 | **<0.001** |
| Ecolabel Total Range [0] | 1.15 | 0.66 – 1.98 | 0.627 |
| Ecolabel Total Range [1] | 1.40 | 0.87 – 2.24 | 0.167 |
| Ecolabel Total Range [2] | 1.06 | 0.67 – 1.66 | 0.808 |
| Ecolabel Total Range [3] | 1.27 | 0.80 – 2.03 | 0.316 |
| Gender [Male] | 0.54 | 0.48 – 0.62 | **<0.001** |
| Gender [Prefer to self-identify] | 1.41 | 0.43 – 4.62 | 0.570 |
| Household Income [Between £25K and £39K] | 0.86 | 0.75 – 1.00 | **0.049** |
| Household Income [Less than £25K] | 0.82 | 0.70 – 0.96 | **0.014** |
| Household Income [Prefer not to say] | 1.12 | 0.84 – 1.49 | 0.424 |
| Device [non-mobile] | 1.09 | 0.95 – 1.25 | 0.224 |
| Age [35-49] | 1.14 | 0.97 – 1.35 | 0.123 |
| Age [50-64] | 1.20 | 1.01 – 1.43 | **0.043** |
| Age [65+] | 1.18 | 0.97 – 1.43 | 0.089 |
| Education Status [Apprenticeship] | 2.03 | 0.77 – 5.33 | 0.153 |
| Education Status [Level 2 qualifications 5 or more GCSEs (A* to C or 9 to 4), O levels (passes), CSEs (grade 1), School Certification, 1 A level, 2 to 3 AS levels, VCEs, Intermediate or Higher Diploma, Welsh Baccalaureate Intermediate Diploma, NVQ level 2, Intermediate GNVQ, City and Guilds Craft, BTEC First or General Diploma, RSA Diploma] | 1.70 | 1.01 – 2.85 | **0.044** |
| Education Status [Level 3 qualifications 2 or more A levels or VCEs, 4 or more AS levels, Higher School Certificate, Progression or Advanced Diploma, Welsh Baccalaureate Advance Diploma, NVQ level 3; Advanced GNVQ, City and Guilds Advanced Craft, ONC, OND, BTEC National, RSA Advanced Diploma] | 2.09 | 1.21 – 3.61 | **0.008** |
| Education Status [Level 4 qualifications or above  degree (BA, BSc), higher degree (MA, PhD, PGCE), NVQ level 4 to 5, HNC, HND, RSA Higher Diploma, BTEC Higher level, professional qualifications (for example, teaching, nursing, accountancy)] | 2.71 | 1.70 – 4.31 | **<0.001** |
| Education Status [No qualifications] | 0.93 | 0.41 – 2.10 | 0.855 |
| Education Status [Other  vocational or work-related qualifications, other qualifications achieved in England or Wales, qualifications achieved outside England or Wales (equivalent not stated or unknown)] | 0.94 | 0.24 – 3.75 | 0.935 |
| Ecolabel Total Range [0] x Education Status [Apprenticeship] | 0.96 | 0.25 – 3.73 | 0.959 |
| Ecolabel Total Range [1] x Education Status [Apprenticeship] | 0.43 | 0.13 – 1.45 | 0.174 |
| Ecolabel Total Range [2] x Education Status [Apprenticeship] | 0.80 | 0.26 – 2.43 | 0.694 |
| Ecolabel Total Range [3] x Education Status [Apprenticeship] | 0.57 | 0.17 – 1.94 | 0.372 |
| Ecolabel Total Range [0] x Education Status [Level 2 qualifications 5 or more GCSEs (A* to C or 9 to 4), O levels (passes), CSEs (grade 1), School Certification, 1 A level, 2 to 3 AS levels, VCEs, Intermediate or Higher Diploma, Welsh Baccalaureate Intermediate Diploma, NVQ level 2, Intermediate GNVQ, City and Guilds Craft, BTEC First or General Diploma, RSA Diploma] | 0.53 | 0.26 – 1.10 | 0.090 |
| Ecolabel Total Range [1] x Education Status [Level 2 qualifications 5 or more GCSEs (A* to C or 9 to 4), O levels (passes), CSEs (grade 1), School Certification, 1 A level, 2 to 3 AS levels, VCEs, Intermediate or Higher Diploma, Welsh Baccalaureate Intermediate Diploma, NVQ level 2, Intermediate GNVQ, City and Guilds Craft, BTEC First or General Diploma, RSA Diploma] | 0.43 | 0.23 – 0.80 | **0.007** |
| Ecolabel Total Range [2] x Education Status [Level 2 qualifications 5 or more GCSEs (A* to C or 9 to 4), O levels (passes), CSEs (grade 1), School Certification, 1 A level, 2 to 3 AS levels, VCEs, Intermediate or Higher Diploma, Welsh Baccalaureate Intermediate Diploma, NVQ level 2, Intermediate GNVQ, City and Guilds Craft, BTEC First or General Diploma, RSA Diploma] | 0.81 | 0.45 – 1.45 | 0.472 |
| Ecolabel Total Range [3] x Education Status [Level 2 qualifications 5 or more GCSEs (A* to C or 9 to 4), O levels (passes), CSEs (grade 1), School Certification, 1 A level, 2 to 3 AS levels, VCEs, Intermediate or Higher Diploma, Welsh Baccalaureate Intermediate Diploma, NVQ level 2, Intermediate GNVQ, City and Guilds Craft, BTEC First or General Diploma, RSA Diploma] | 0.66 | 0.36 – 1.22 | 0.190 |
| Ecolabel Total Range [0] x Education Status [Level 3 qualifications 2 or more A levels or VCEs, 4 or more AS levels, Higher School Certificate, Progression or Advanced Diploma, Welsh Baccalaureate Advance Diploma, NVQ level 3; Advanced GNVQ, City and Guilds Advanced Craft, ONC, OND, BTEC National, RSA Advanced Diploma] | 0.59 | 0.28 – 1.25 | 0.167 |
| Ecolabel Total Range [1] x Education Status [Level 3 qualifications 2 or more A levels or VCEs, 4 or more AS levels, Higher School Certificate, Progression or Advanced Diploma, Welsh Baccalaureate Advance Diploma, NVQ level 3; Advanced GNVQ, City and Guilds Advanced Craft, ONC, OND, BTEC National, RSA Advanced Diploma] | 0.59 | 0.31 – 1.13 | 0.112 |
| Ecolabel Total Range [2] x Education Status [Level 3 qualifications 2 or more A levels or VCEs, 4 or more AS levels, Higher School Certificate, Progression or Advanced Diploma, Welsh Baccalaureate Advance Diploma, NVQ level 3; Advanced GNVQ, City and Guilds Advanced Craft, ONC, OND, BTEC National, RSA Advanced Diploma] | 0.74 | 0.40 – 1.37 | 0.337 |
| Ecolabel Total Range [3] x Education Status [Level 3 qualifications 2 or more A levels or VCEs, 4 or more AS levels, Higher School Certificate, Progression or Advanced Diploma, Welsh Baccalaureate Advance Diploma, NVQ level 3; Advanced GNVQ, City and Guilds Advanced Craft, ONC, OND, BTEC National, RSA Advanced Diploma] | 0.58 | 0.30 – 1.11 | 0.102 |
| Ecolabel Total Range [0] x Education Status [Level 4 qualifications or above  degree (BA, BSc), higher degree (MA, PhD, PGCE), NVQ level 4 to 5, HNC, HND, RSA Higher Diploma, BTEC Higher level, professional qualifications (for example, teaching, nursing, accountancy)] | 0.50 | 0.26 – 0.95 | **0.035** |
| Ecolabel Total Range [1] x Education Status [Level 4 qualifications or above  degree (BA, BSc), higher degree (MA, PhD, PGCE), NVQ level 4 to 5, HNC, HND, RSA Higher Diploma, BTEC Higher level, professional qualifications (for example, teaching, nursing, accountancy)] | 0.54 | 0.31 – 0.94 | **0.030** |
| Ecolabel Total Range [2] x Education Status [Level 4 qualifications or above  degree (BA, BSc), higher degree (MA, PhD, PGCE), NVQ level 4 to 5, HNC, HND, RSA Higher Diploma, BTEC Higher level, professional qualifications (for example, teaching, nursing, accountancy)] | 0.71 | 0.42 – 1.19 | 0.190 |
| Ecolabel Total Range [3] x Education Status [Level 4 qualifications or above  degree (BA, BSc), higher degree (MA, PhD, PGCE), NVQ level 4 to 5, HNC, HND, RSA Higher Diploma, BTEC Higher level, professional qualifications (for example, teaching, nursing, accountancy)] | 0.58 | 0.34 – 0.99 | **0.048** |
| Ecolabel Total Range [0] x Education Status [No qualifications] | 1.00 | 0.30 – 3.28 | 0.994 |
| Ecolabel Total Range [1] x Education Status [No qualifications] | 0.64 | 0.23 – 1.77 | 0.391 |
| Ecolabel Total Range [2] x Education Status [No qualifications] | 1.01 | 0.39 – 2.59 | 0.986 |
| Ecolabel Total Range [3] x Education Status [No qualifications] | 1.38 | 0.54 – 3.53 | 0.504 |
| Ecolabel Total Range [0] x Education Status [Other vocational or work-related qualifications, other qualifications achieved in England or Wales, qualifications achieved outside England or Wales (equivalent not stated or unknown)] | 1.87 | 0.33 – 10.59 | 0.478 |
| Ecolabel Total Range [1] x Education Status [Other vocational or work-related qualifications, other qualifications achieved in England or Wales, qualifications achieved outside England or Wales (equivalent not stated or unknown)] | 1.34 | 0.27 – 6.65 | 0.718 |
| Ecolabel Total Range [2] x Education Status [Other vocational or work-related qualifications, other qualifications achieved in England or Wales, qualifications achieved outside England or Wales (equivalent not stated or unknown)] | 1.84 | 0.40 – 8.41 | 0.431 |
| Ecolabel Total Range [3] x Education Status [Other vocational or work-related qualifications, other qualifications achieved in England or Wales, qualifications achieved outside England or Wales (equivalent not stated or unknown)] | 3.34 | 0.63 – 17.56 | 0.155 |
| **Random Effects** | | | |
| σ^2^ | 3.29 | | |
| τ_00_ _ResponseId_ | 0.66 | | |
| ICC | 0.17 | | |
| N _ResponseId_ | 2298 | | |
| Observations | 11490 | | |
| Marginal R^2^ / Conditional R^2^ | 0.042 / 0.202 | | |

#### Household income

*Table 13a: Outcome measure: Low/medium*

|  |  | | |
| --- | --- | --- | --- |
| *Predictors* | *Odds Ratios* | *CI* | *p* |
| (Intercept) | 3.39 | 2.67 – 4.29 | **<0.001** |
| Ecolabel Total Range [0] | 0.89 | 0.67 – 1.17 | 0.408 |
| Ecolabel Total Range [1] | 0.93 | 0.73 – 1.18 | 0.565 |
| Ecolabel Total Range [2] | 0.95 | 0.76 – 1.19 | 0.650 |
| Ecolabel Total Range [3] | 1.16 | 0.90 – 1.48 | 0.251 |
| Gender [Male] | 0.73 | 0.67 – 0.79 | **<0.001** |
| Gender [Prefer to self-identify] | 0.91 | 0.35 – 2.32 | 0.837 |
| Education Status [Apprenticeship] | 0.88 | 0.67 – 1.15 | 0.359 |
| Education Status [Level 2 qualifications 5 or more GCSEs (A* to C or 9 to 4), O levels (passes), CSEs (grade 1), School Certification, 1 A level, 2 to 3 AS levels, VCEs, Intermediate or Higher Diploma, Welsh Baccalaureate Intermediate Diploma, NVQ level 2, Intermediate GNVQ, City and Guilds Craft, BTEC First or General Diploma, RSA Diploma] | 0.98 | 0.86 – 1.12 | 0.796 |
| Education Status [Level 3 qualifications 2 or more A levels or VCEs, 4 or more AS levels, Higher School Certificate, Progression or Advanced Diploma, Welsh Baccalaureate Advance Diploma, NVQ level 3; Advanced GNVQ, City and Guilds Advanced Craft, ONC, OND, BTEC National, RSA Advanced Diploma] | 1.08 | 0.94 – 1.25 | 0.288 |
| Education Status [Level 4 qualifications or above  degree (BA, BSc), higher degree (MA, PhD, PGCE), NVQ level 4 to 5, HNC, HND, RSA Higher Diploma, BTEC Higher level, professional qualifications (for example, teaching, nursing, accountancy)] | 1.20 | 1.06 – 1.37 | **0.004** |
| Education Status [No qualifications] | 0.94 | 0.76 – 1.16 | 0.537 |
| Education Status [Other  vocational or work-related qualifications, other qualifications achieved in England or Wales, qualifications achieved outside England or Wales (equivalent not stated or unknown)] | 1.18 | 0.82 – 1.70 | 0.362 |
| Device [non-mobile] | 0.93 | 0.84 – 1.02 | 0.133 |
| Age [35-49] | 0.74 | 0.66 – 0.84 | **<0.001** |
| Age [50-64] | 0.68 | 0.60 – 0.77 | **<0.001** |
| Age [65+] | 0.58 | 0.51 – 0.66 | **<0.001** |
| Household Income [Between £25K and £39K] | 1.02 | 0.75 – 1.37 | 0.918 |
| Household Income [Less than £25K] | 1.02 | 0.75 – 1.39 | 0.881 |
| Household Income [Prefer not to say] | 0.90 | 0.48 – 1.70 | 0.751 |
| Ecolabel Total Range [0] x Household Income [Between £25K and £39K] | 0.76 | 0.50 – 1.14 | 0.182 |
| Ecolabel Total Range [1] x Household Income [Between £25K and £39K] | 0.91 | 0.64 – 1.30 | 0.609 |
| Ecolabel Total Range [2] x Household Income [Between £25K and £39K] | 1.00 | 0.71 – 1.40 | 0.985 |
| Ecolabel Total Range [3] x Household Income [Between £25K and £39K] | 0.75 | 0.52 – 1.08 | 0.121 |
| Ecolabel Total Range [0] x Household Income [Less than £25K] | 0.78 | 0.51 – 1.19 | 0.255 |
| Ecolabel Total Range [1] x Household Income [Less than £25K] | 1.02 | 0.70 – 1.48 | 0.927 |
| Ecolabel Total Range [2] x Household Income [Less than £25K] | 0.98 | 0.69 – 1.39 | 0.900 |
| Ecolabel Total Range [3] x Household Income [Less than £25K] | 0.76 | 0.52 – 1.10 | 0.143 |
| Ecolabel Total Range [0] x Household Income [Prefer not to say] | 1.40 | 0.57 – 3.46 | 0.464 |
| Ecolabel Total Range [1] x Household Income [Prefer not to say] | 1.18 | 0.55 – 2.52 | 0.673 |
| Ecolabel Total Range [2] x Household Income [Prefer not to say] | 1.01 | 0.50 – 2.07 | 0.971 |
| Ecolabel Total Range [3] x Household Income [Prefer not to say] | 1.28 | 0.60 – 2.76 | 0.524 |
| **Random Effects** | | | |
| σ^2^ | 3.29 | | |
| τ_00_ _ResponseId_ | 0.10 | | |
| ICC | 0.03 | | |
| N _ResponseId_ | 2298 | | |
| Observations | 11490 | | |
| Marginal R^2^ / Conditional R^2^ | 0.033 / 0.060 | | |

*Table 13b: Outcome measure: Low*

|  |  | | |
| --- | --- | --- | --- |
| *Predictors* | *Odds Ratios* | *CI* | *p* |
| (Intercept) | 0.27 | 0.20 – 0.36 | **<0.001** |
| Ecolabel Total Range [0] | 0.63 | 0.46 – 0.88 | **0.007** |
| Ecolabel Total Range [1] | 0.74 | 0.56 – 0.97 | **0.028** |
| Ecolabel Total Range [2] | 0.73 | 0.56 – 0.95 | **0.017** |
| Ecolabel Total Range [3] | 0.82 | 0.62 – 1.08 | 0.164 |
| Gender [Male] | 0.55 | 0.48 – 0.62 | **<0.001** |
| Gender [Prefer to self-identify] | 1.35 | 0.41 – 4.42 | 0.617 |
| Education Status [Apprenticeship] | 1.39 | 0.93 – 2.09 | 0.110 |
| Education Status [Level 2 qualifications 5 or more GCSEs (A* to C or 9 to 4), O levels (passes), CSEs (grade 1), School Certification, 1 A level, 2 to 3 AS levels, VCEs, Intermediate or Higher Diploma, Welsh Baccalaureate Intermediate Diploma, NVQ level 2, Intermediate GNVQ, City and Guilds Craft, BTEC First or General Diploma, RSA Diploma] | 1.12 | 0.92 – 1.37 | 0.246 |
| Education Status [Level 3 qualifications 2 or more A levels or VCEs, 4 or more AS levels, Higher School Certificate, Progression or Advanced Diploma, Welsh Baccalaureate Advance Diploma, NVQ level 3; Advanced GNVQ, City and Guilds Advanced Craft, ONC, OND, BTEC National, RSA Advanced Diploma] | 1.41 | 1.15 – 1.75 | **0.001** |
| Education Status [Level 4 qualifications or above  degree (BA, BSc), higher degree (MA, PhD, PGCE), NVQ level 4 to 5, HNC, HND, RSA Higher Diploma, BTEC Higher level, professional qualifications (for example, teaching, nursing, accountancy)] | 1.74 | 1.45 – 2.08 | **<0.001** |
| Education Status [No qualifications] | 0.93 | 0.67 – 1.28 | 0.649 |
| Education Status [Other  vocational or work-related qualifications, other qualifications achieved in England or Wales, qualifications achieved outside England or Wales (equivalent not stated or unknown)] | 1.65 | 1.01 – 2.71 | **0.046** |
| Device [non-mobile] | 1.09 | 0.95 – 1.25 | 0.211 |
| Age [35-49] | 1.14 | 0.97 – 1.35 | 0.118 |
| Age [50-64] | 1.20 | 1.01 – 1.43 | **0.040** |
| Age [65+] | 1.18 | 0.97 – 1.42 | 0.097 |
| Household Income [Between £25K and £39K] | 0.76 | 0.54 – 1.09 | 0.136 |
| Household Income [Less than £25K] | 0.71 | 0.49 – 1.03 | 0.074 |
| Household Income [Prefer not to say] | 0.60 | 0.27 – 1.34 | 0.214 |
| Ecolabel Total Range [0] x Household Income [Between £25K and £39K] | 1.13 | 0.68 – 1.88 | 0.645 |
| Ecolabel Total Range [1] x Household Income [Between £25K and £39K] | 1.11 | 0.72 – 1.70 | 0.634 |
| Ecolabel Total Range [2] x Household Income [Between £25K and £39K] | 1.29 | 0.87 – 1.93 | 0.209 |
| Ecolabel Total Range [3] x Household Income [Between £25K and £39K] | 1.01 | 0.66 – 1.55 | 0.951 |
| Ecolabel Total Range [0] x Household Income [Less than £25K] | 1.10 | 0.64 – 1.88 | 0.735 |
| Ecolabel Total Range [1] x Household Income [Less than £25K] | 1.20 | 0.77 – 1.89 | 0.424 |
| Ecolabel Total Range [2] x Household Income [Less than £25K] | 1.20 | 0.79 – 1.84 | 0.389 |
| Ecolabel Total Range [3] x Household Income [Less than £25K] | 1.17 | 0.75 – 1.84 | 0.488 |
| Ecolabel Total Range [0] x Household Income [Prefer not to say] | 3.28 | 1.15 – 9.35 | **0.027** |
| Ecolabel Total Range [1] x Household Income [Prefer not to say] | 2.17 | 0.87 – 5.42 | 0.096 |
| Ecolabel Total Range [2] x Household Income [Prefer not to say] | 1.70 | 0.70 – 4.10 | 0.237 |
| Ecolabel Total Range [3] x Household Income [Prefer not to say] | 1.87 | 0.76 – 4.64 | 0.174 |
| **Random Effects** | | | |
| σ^2^ | 3.29 | | |
| τ_00_ _ResponseId_ | 0.66 | | |
| ICC | 0.17 | | |
| N _ResponseId_ | 2298 | | |
| Observations | 11490 | | |
| Marginal R^2^ / Conditional R^2^ | 0.039 / 0.199 | | |

#### Device type

*Table 14a: Outcome measure: Low/medium*

|  | **Meal.Choice.H.0** | | |
| --- | --- | --- | --- |
| *Predictors* | *Odds Ratios* | *CI* | *p* |
| (Intercept) | 3.80 | 3.01 – 4.79 | **<0.001** |
| Ecolabel Total Range [0] | 0.67 | 0.52 – 0.87 | **0.002** |
| Ecolabel Total Range [1] | 0.88 | 0.70 – 1.10 | 0.265 |
| Ecolabel Total Range [2] | 0.89 | 0.72 – 1.11 | 0.307 |
| Ecolabel Total Range [3] | 0.93 | 0.74 – 1.18 | 0.556 |
| Gender [Male] | 0.73 | 0.67 – 0.79 | **<0.001** |
| Gender [Prefer to self-identify] | 0.92 | 0.36 – 2.35 | 0.860 |
| Education Status [Apprenticeship] | 0.88 | 0.67 – 1.16 | 0.364 |
| Education Status [Level 2 qualifications 5 or more GCSEs (A* to C or 9 to 4), O levels (passes), CSEs (grade 1), School Certification, 1 A level, 2 to 3 AS levels, VCEs, Intermediate or Higher Diploma, Welsh Baccalaureate Intermediate Diploma, NVQ level 2, Intermediate GNVQ, City and Guilds Craft, BTEC First or General Diploma, RSA Diploma] | 0.98 | 0.86 – 1.12 | 0.790 |
| Education Status [Level 3 qualifications 2 or more A levels or VCEs, 4 or more AS levels, Higher School Certificate, Progression or Advanced Diploma, Welsh Baccalaureate Advance Diploma, NVQ level 3; Advanced GNVQ, City and Guilds Advanced Craft, ONC, OND, BTEC National, RSA Advanced Diploma] | 1.08 | 0.94 – 1.25 | 0.289 |
| Education Status [Level 4 qualifications or above  degree (BA, BSc), higher degree (MA, PhD, PGCE), NVQ level 4 to 5, HNC, HND, RSA Higher Diploma, BTEC Higher level, professional qualifications (for example, teaching, nursing, accountancy)] | 1.20 | 1.06 – 1.36 | **0.004** |
| Education Status [No qualifications] | 0.93 | 0.76 – 1.15 | 0.530 |
| Education Status [Other  vocational or work-related qualifications, other qualifications achieved in England or Wales, qualifications achieved outside England or Wales (equivalent not stated or unknown)] | 1.18 | 0.82 – 1.70 | 0.361 |
| Household Income [Between £25K and £39K] | 0.90 | 0.82 – 1.00 | 0.055 |
| Household Income [Less than £25K] | 0.93 | 0.83 – 1.04 | 0.229 |
| Household Income [Prefer not to say] | 1.03 | 0.84 – 1.28 | 0.761 |
| Age [35-49] | 0.74 | 0.66 – 0.84 | **<0.001** |
| Age [50-64] | 0.68 | 0.60 – 0.77 | **<0.001** |
| Age [65+] | 0.58 | 0.50 – 0.66 | **<0.001** |
| Device [non-mobile] | 0.84 | 0.65 – 1.09 | 0.186 |
| Ecolabel Total Range [0] x Device [non-mobile] | 1.29 | 0.91 – 1.81 | 0.149 |
| Ecolabel Total Range [1] x Device [non-mobile] | 1.08 | 0.80 – 1.46 | 0.630 |
| Ecolabel Total Range [2] x Device [non-mobile] | 1.10 | 0.82 – 1.46 | 0.532 |
| Ecolabel Total Range [3] x Device [non-mobile] | 1.10 | 0.81 – 1.48 | 0.554 |
| **Random Effects** | | | |
| σ^2^ | 3.29 | | |
| τ_00_ _ResponseId_ | 0.09 | | |
| ICC | 0.03 | | |
| N _ResponseId_ | 2298 | | |
| Observations | 11490 | | |
| Marginal R^2^ / Conditional R^2^ | 0.032 / 0.058 | | |

*Table 14b: Outcome measure: Low*

|  |  | | |
| --- | --- | --- | --- |
| *Predictors* | *Odds Ratios* | *CI* | *p* |
| (Intercept) | 0.24 | 0.18 – 0.32 | **<0.001** |
| Ecolabel Total Range [0] | 0.72 | 0.53 – 0.99 | **0.042** |
| Ecolabel Total Range [1] | 0.82 | 0.63 – 1.07 | 0.144 |
| Ecolabel Total Range [2] | 0.91 | 0.71 – 1.17 | 0.448 |
| Ecolabel Total Range [3] | 0.89 | 0.68 – 1.16 | 0.390 |
| Gender [Male] | 0.55 | 0.48 – 0.62 | **<0.001** |
| Gender [Prefer to self-identify] | 1.37 | 0.42 – 4.48 | 0.598 |
| Education Status [Apprenticeship] | 1.39 | 0.92 – 2.08 | 0.115 |
| Education Status [Level 2 qualifications 5 or more GCSEs (A* to C or 9 to 4), O levels (passes), CSEs (grade 1), School Certification, 1 A level, 2 to 3 AS levels, VCEs, Intermediate or Higher Diploma, Welsh Baccalaureate Intermediate Diploma, NVQ level 2, Intermediate GNVQ, City and Guilds Craft, BTEC First or General Diploma, RSA Diploma] | 1.12 | 0.92 – 1.37 | 0.253 |
| Education Status [Level 3 qualifications 2 or more A levels or VCEs, 4 or more AS levels, Higher School Certificate, Progression or Advanced Diploma, Welsh Baccalaureate Advance Diploma, NVQ level 3; Advanced GNVQ, City and Guilds Advanced Craft, ONC, OND, BTEC National, RSA Advanced Diploma] | 1.41 | 1.14 – 1.74 | **0.001** |
| Education Status [Level 4 qualifications or above  degree (BA, BSc), higher degree (MA, PhD, PGCE), NVQ level 4 to 5, HNC, HND, RSA Higher Diploma, BTEC Higher level, professional qualifications (for example, teaching, nursing, accountancy)] | 1.73 | 1.44 – 2.07 | **<0.001** |
| Education Status [No qualifications] | 0.92 | 0.67 – 1.28 | 0.635 |
| Education Status [Other  vocational or work-related qualifications, other qualifications achieved in England or Wales, qualifications achieved outside England or Wales (equivalent not stated or unknown)] | 1.66 | 1.01 – 2.71 | **0.044** |
| Household Income [Between £25K and £39K] | 0.86 | 0.75 – 1.00 | **0.046** |
| Household Income [Less than £25K] | 0.82 | 0.70 – 0.96 | **0.016** |
| Household Income [Prefer not to say] | 1.13 | 0.85 – 1.51 | 0.385 |
| Age [35-49] | 1.14 | 0.97 – 1.35 | 0.119 |
| Age [50-64] | 1.20 | 1.00 – 1.42 | **0.045** |
| Age [65+] | 1.17 | 0.97 – 1.42 | 0.100 |
| Device [non-mobile] | 1.15 | 0.85 – 1.55 | 0.375 |
| Ecolabel Total Range [0] x Device [non-mobile] | 0.97 | 0.64 – 1.49 | 0.900 |
| Ecolabel Total Range [1] x Device [non-mobile] | 1.00 | 0.70 – 1.43 | 0.991 |
| Ecolabel Total Range [2] x Device [non-mobile] | 0.87 | 0.63 – 1.22 | 0.433 |
| Ecolabel Total Range [3] x Device [non-mobile] | 0.98 | 0.68 – 1.40 | 0.903 |
| **Random Effects** | | | |
| σ^2^ | 3.29 | | |
| τ_00_ _ResponseId_ | 0.65 | | |
| ICC | 0.17 | | |
| N _ResponseId_ | 2298 | | |
| Observations | 11490 | | |
| Marginal R^2^ / Conditional R^2^ | 0.038 / 0.197 | | |

#### Meat eating frequency

*Table 15a: Outcome measure: Low/medium*

|  |  | | |
| --- | --- | --- | --- |
| *Predictors* | *Odds Ratios* | *CI* | *p* |
| (Intercept) | 5.10 | 3.33 – 7.80 | **<0.001** |
| Ecolabel Total Range [0] | 0.77 | 0.44 – 1.32 | 0.338 |
| Ecolabel Total Range [1] | 0.90 | 0.56 – 1.45 | 0.666 |
| Ecolabel Total Range [2] | 0.87 | 0.55 – 1.37 | 0.551 |
| Ecolabel Total Range [3] | 0.76 | 0.47 – 1.22 | 0.254 |
| Gender [Male] | 0.74 | 0.68 – 0.81 | **<0.001** |
| Gender [Prefer to self-identify] | 1.49 | 0.47 – 4.79 | 0.500 |
| Education Status [Apprenticeship] | 0.91 | 0.69 – 1.19 | 0.479 |
| Education Status [Level 2 qualifications 5 or more GCSEs (A* to C or 9 to 4), O levels (passes), CSEs (grade 1), School Certification, 1 A level, 2 to 3 AS levels, VCEs, Intermediate or Higher Diploma, Welsh Baccalaureate Intermediate Diploma, NVQ level 2, Intermediate GNVQ, City and Guilds Craft, BTEC First or General Diploma, RSA Diploma] | 1.00 | 0.87 – 1.14 | 0.990 |
| Education Status [Level 3 qualifications 2 or more A levels or VCEs, 4 or more AS levels, Higher School Certificate, Progression or Advanced Diploma, Welsh Baccalaureate Advance Diploma, NVQ level 3; Advanced GNVQ, City and Guilds Advanced Craft, ONC, OND, BTEC National, RSA Advanced Diploma] | 1.11 | 0.95 – 1.28 | 0.180 |
| Education Status [Level 4 qualifications or above  degree (BA, BSc), higher degree (MA, PhD, PGCE), NVQ level 4 to 5, HNC, HND, RSA Higher Diploma, BTEC Higher level, professional qualifications (for example, teaching, nursing, accountancy)] | 1.22 | 1.08 – 1.38 | **0.002** |
| Education Status [No qualifications] | 0.92 | 0.74 – 1.14 | 0.446 |
| Education Status [Other  vocational or work-related qualifications, other qualifications achieved in England or Wales, qualifications achieved outside England or Wales (equivalent not stated or unknown)] | 1.21 | 0.84 – 1.73 | 0.308 |
| Household Income [Between £25K and £39K] | 0.90 | 0.81 – 0.99 | **0.037** |
| Household Income [Less than £25K] | 0.92 | 0.83 – 1.03 | 0.165 |
| Household Income [Prefer not to say] | 1.02 | 0.82 – 1.26 | 0.859 |
| Device [non-mobile] | 0.93 | 0.84 – 1.02 | 0.134 |
| Age [35-49] | 0.73 | 0.65 – 0.83 | **<0.001** |
| Age [50-64] | 0.67 | 0.59 – 0.76 | **<0.001** |
| Age [65+] | 0.57 | 0.50 – 0.65 | **<0.001** |
| Total Meat Eating Frequency Score | 0.94 | 0.88 – 1.00 | 0.061 |
| Ecolabel Total Range [0] x Total Meat Eating Frequency Score | 1.00 | 0.92 – 1.09 | 0.986 |
| Ecolabel Total Range [1] x Total Meat Eating Frequency Score | 1.00 | 0.93 – 1.08 | 0.957 |
| Ecolabel Total Range [2] x Total Meat Eating Frequency Score | 1.01 | 0.94 – 1.09 | 0.706 |
| Ecolabel Total Range [3] x Total Meat Eating Frequency Score | 1.04 | 0.97 – 1.13 | 0.257 |
| **Random Effects** | | | |
| σ^2^ | 3.29 | | |
| τ_00_ _ResponseId_ | 0.09 | | |
| ICC | 0.03 | | |
| N _ResponseId_ | 2289 | | |
| Observations | 11445 | | |
| Marginal R^2^ / Conditional R^2^ | 0.034 / 0.059 | | |

*Table 15b: Outcome measure: Low*

|  |  | | |
| --- | --- | --- | --- |
| *Predictors* | *Odds Ratios* | *CI* | *p* |
| (Intercept) | 0.58 | 0.35 – 0.96 | **0.034** |
| Ecolabel Total Range [0] | 0.65 | 0.34 – 1.25 | 0.198 |
| Ecolabel Total Range [1] | 0.80 | 0.46 – 1.38 | 0.418 |
| Ecolabel Total Range [2] | 0.82 | 0.49 – 1.38 | 0.453 |
| Ecolabel Total Range [3] | 0.69 | 0.40 – 1.20 | 0.189 |
| Gender [Male] | 0.58 | 0.51 – 0.65 | **<0.001** |
| Gender [Prefer to self-identify] | 1.88 | 0.54 – 6.61 | 0.324 |
| Education Status [Apprenticeship] | 1.47 | 0.98 – 2.19 | 0.061 |
| Education Status [Level 2 qualifications 5 or more GCSEs (A* to C or 9 to 4), O levels (passes), CSEs (grade 1), School Certification, 1 A level, 2 to 3 AS levels, VCEs, Intermediate or Higher Diploma, Welsh Baccalaureate Intermediate Diploma, NVQ level 2, Intermediate GNVQ, City and Guilds Craft, BTEC First or General Diploma, RSA Diploma] | 1.18 | 0.97 – 1.43 | 0.104 |
| Education Status [Level 3 qualifications 2 or more A levels or VCEs, 4 or more AS levels, Higher School Certificate, Progression or Advanced Diploma, Welsh Baccalaureate Advance Diploma, NVQ level 3; Advanced GNVQ, City and Guilds Advanced Craft, ONC, OND, BTEC National, RSA Advanced Diploma] | 1.49 | 1.21 – 1.84 | **<0.001** |
| Education Status [Level 4 qualifications or above  degree (BA, BSc), higher degree (MA, PhD, PGCE), NVQ level 4 to 5, HNC, HND, RSA Higher Diploma, BTEC Higher level, professional qualifications (for example, teaching, nursing, accountancy)] | 1.79 | 1.49 – 2.14 | **<0.001** |
| Education Status [No qualifications] | 0.90 | 0.65 – 1.24 | 0.506 |
| Education Status [Other  vocational or work-related qualifications, other qualifications achieved in England or Wales, qualifications achieved outside England or Wales (equivalent not stated or unknown)] | 1.74 | 1.08 – 2.83 | **0.024** |
| Household Income [Between £25K and £39K] | 0.84 | 0.72 – 0.96 | **0.013** |
| Household Income [Less than £25K] | 0.78 | 0.67 – 0.91 | **0.002** |
| Household Income [Prefer not to say] | 1.02 | 0.77 – 1.36 | 0.886 |
| Device [non-mobile] | 1.09 | 0.96 – 1.25 | 0.182 |
| Age [35-49] | 1.10 | 0.93 – 1.29 | 0.272 |
| Age [50-64] | 1.13 | 0.95 – 1.34 | 0.167 |
| Age [65+] | 1.12 | 0.93 – 1.36 | 0.226 |
| Total Meat Eating Frequency Score | 0.86 | 0.80 – 0.93 | **<0.001** |
| Ecolabel Total Range [0] x Total Meat Eating Frequency Score | 1.01 | 0.91 – 1.13 | 0.832 |
| Ecolabel Total Range [1] x Total Meat Eating Frequency Score | 1.00 | 0.92 – 1.10 | 0.934 |
| Ecolabel Total Range [2] x Total Meat Eating Frequency Score | 1.00 | 0.92 – 1.09 | 0.926 |
| Ecolabel Total Range [3] x Total Meat Eating Frequency Score | 1.04 | 0.95 – 1.14 | 0.375 |
| **Random Effects** | | | |
| σ^2^ | 3.29 | | |
| τ_00_ _ResponseId_ | 0.59 | | |
| ICC | 0.15 | | |
| N _ResponseId_ | 2289 | | |
| Observations | 11445 | | |
| Marginal R^2^ / Conditional R^2^ | 0.055 / 0.198 | | |

#### Cafeteria usage

*Table 16a: Outcome measure: Low/medium*

|  |  | | |
| --- | --- | --- | --- |
| *Predictors* | *Odds Ratios* | *CI* | *p* |
| (Intercept) | 4.10 | 2.93 – 5.75 | **<0.001** |
| Ecolabel Total Range [0] | 0.73 | 0.47 – 1.11 | 0.139 |
| Ecolabel Total Range [1] | 0.83 | 0.58 – 1.21 | 0.337 |
| Ecolabel Total Range [2] | 0.98 | 0.69 – 1.39 | 0.910 |
| Ecolabel Total Range [3] | 0.94 | 0.65 – 1.36 | 0.738 |
| Gender [Male] | 0.73 | 0.66 – 0.79 | **<0.001** |
| Gender [Prefer to self-identify] | 1.60 | 0.42 – 6.15 | 0.495 |
| Education Status [Apprenticeship] | 0.90 | 0.69 – 1.18 | 0.448 |
| Education Status [Level 2 qualifications 5 or more GCSEs (A* to C or 9 to 4), O levels (passes), CSEs (grade 1), School Certification, 1 A level, 2 to 3 AS levels, VCEs, Intermediate or Higher Diploma, Welsh Baccalaureate Intermediate Diploma, NVQ level 2, Intermediate GNVQ, City and Guilds Craft, BTEC First or General Diploma, RSA Diploma] | 0.99 | 0.87 – 1.14 | 0.906 |
| Education Status [Level 3 qualifications 2 or more A levels or VCEs, 4 or more AS levels, Higher School Certificate, Progression or Advanced Diploma, Welsh Baccalaureate Advance Diploma, NVQ level 3; Advanced GNVQ, City and Guilds Advanced Craft, ONC, OND, BTEC National, RSA Advanced Diploma] | 1.09 | 0.94 – 1.27 | 0.239 |
| Education Status [Level 4 qualifications or above  degree (BA, BSc), higher degree (MA, PhD, PGCE), NVQ level 4 to 5, HNC, HND, RSA Higher Diploma, BTEC Higher level, professional qualifications (for example, teaching, nursing, accountancy)] | 1.21 | 1.07 – 1.38 | **0.003** |
| Education Status [No qualifications] | 0.93 | 0.75 – 1.15 | 0.521 |
| Education Status [Other  vocational or work-related qualifications, other qualifications achieved in England or Wales, qualifications achieved outside England or Wales (equivalent not stated or unknown)] | 1.18 | 0.82 – 1.71 | 0.380 |
| Household Income [Between £25K and £39K] | 0.90 | 0.81 – 1.00 | **0.042** |
| Household Income [Less than £25K] | 0.92 | 0.82 – 1.03 | 0.148 |
| Household Income [Prefer not to say] | 1.04 | 0.83 – 1.29 | 0.742 |
| Device [non-mobile] | 0.93 | 0.84 – 1.02 | 0.129 |
| Age [35-49] | 0.74 | 0.65 – 0.84 | **<0.001** |
| Age [50-64] | 0.68 | 0.60 – 0.77 | **<0.001** |
| Age [65+] | 0.57 | 0.50 – 0.66 | **<0.001** |
| Cafeteria Usage [3-4 days a week] | 0.79 | 0.46 – 1.35 | 0.386 |
| Cafeteria Usage [5-6 days a week] | 0.63 | 0.26 – 1.53 | 0.304 |
| Cafeteria Usage [Less than once a week] | 0.82 | 0.57 – 1.19 | 0.294 |
| Cafeteria Usage [Never] | 0.90 | 0.63 – 1.31 | 0.591 |
| Ecolabel Total Range [0] x Cafeteria Usage [3-4 days a week] | 1.61 | 0.75 – 3.46 | 0.222 |
| Ecolabel Total Range [1] x Cafeteria Usage [3-4 days a week] | 0.93 | 0.49 – 1.77 | 0.825 |
| Ecolabel Total Range [2] x Cafeteria Usage [3-4 days a week] | 1.18 | 0.64 – 2.19 | 0.595 |
| Ecolabel Total Range [3] x Cafeteria Usage [3-4 days a week] | 1.04 | 0.54 – 2.01 | 0.902 |
| Ecolabel Total Range [0] x Cafeteria Usage [5-6 days a week] | 4.16 | 0.87 – 19.88 | 0.074 |
| Ecolabel Total Range [1] x Cafeteria Usage [5-6 days a week] | 0.96 | 0.32 – 2.87 | 0.943 |
| Ecolabel Total Range [2] x Cafeteria Usage [5-6 days a week] | 1.06 | 0.37 – 3.02 | 0.912 |
| Ecolabel Total Range [3] x Cafeteria Usage [5-6 days a week] | 1.06 | 0.36 – 3.14 | 0.919 |
| Ecolabel Total Range [0] x Cafeteria Usage [Less than once a week] | 0.95 | 0.57 – 1.59 | 0.857 |
| Ecolabel Total Range [1] x Cafeteria Usage [Less than once a week] | 1.15 | 0.74 – 1.79 | 0.539 |
| Ecolabel Total Range [2] x Cafeteria Usage [Less than once a week] | 0.94 | 0.62 – 1.44 | 0.786 |
| Ecolabel Total Range [3] x Cafeteria Usage [Less than once a week] | 1.17 | 0.75 – 1.83 | 0.500 |
| Ecolabel Total Range [0] x Cafeteria Usage [Never] | 1.12 | 0.68 – 1.86 | 0.660 |
| Ecolabel Total Range [1] x Cafeteria Usage [Never] | 1.16 | 0.74 – 1.80 | 0.520 |
| Ecolabel Total Range [2] x Cafeteria Usage [Never] | 0.95 | 0.63 – 1.45 | 0.818 |
| Ecolabel Total Range [3] x Cafeteria Usage [Never] | 0.98 | 0.63 – 1.53 | 0.940 |
| **Random Effects** | | | |
| σ^2^ | 3.29 | | |
| τ_00_ _ResponseId_ | 0.10 | | |
| ICC | 0.03 | | |
| N _ResponseId_ | 2270 | | |
| Observations | 11350 | | |
| Marginal R^2^ / Conditional R^2^ | 0.035 / 0.063 | | |

*Table 16b: Outcome measure: Low*

|  |  | | |
| --- | --- | --- | --- |
| *Predictors* | *Odds Ratios* | *CI* | *p* |
| (Intercept) | 0.24 | 0.16 – 0.36 | **<0.001** |
| Ecolabel Total Range [0] | 0.91 | 0.55 – 1.49 | 0.697 |
| Ecolabel Total Range [1] | 0.85 | 0.55 – 1.30 | 0.449 |
| Ecolabel Total Range [2] | 0.98 | 0.66 – 1.45 | 0.907 |
| Ecolabel Total Range [3] | 0.92 | 0.60 – 1.40 | 0.692 |
| Gender [Male] | 0.55 | 0.48 – 0.62 | **<0.001** |
| Gender [Prefer to self-identify] | 1.34 | 0.29 – 6.17 | 0.706 |
| Education Status [Apprenticeship] | 1.41 | 0.94 – 2.12 | 0.099 |
| Education Status [Level 2 qualifications 5 or more GCSEs (A* to C or 9 to 4), O levels (passes), CSEs (grade 1), School Certification, 1 A level, 2 to 3 AS levels, VCEs, Intermediate or Higher Diploma, Welsh Baccalaureate Intermediate Diploma, NVQ level 2, Intermediate GNVQ, City and Guilds Craft, BTEC First or General Diploma, RSA Diploma] | 1.14 | 0.93 – 1.39 | 0.208 |
| Education Status [Level 3 qualifications 2 or more A levels or VCEs, 4 or more AS levels, Higher School Certificate, Progression or Advanced Diploma, Welsh Baccalaureate Advance Diploma, NVQ level 3; Advanced GNVQ, City and Guilds Advanced Craft, ONC, OND, BTEC National, RSA Advanced Diploma] | 1.44 | 1.16 – 1.78 | **0.001** |
| Education Status [Level 4 qualifications or above  degree (BA, BSc), higher degree (MA, PhD, PGCE), NVQ level 4 to 5, HNC, HND, RSA Higher Diploma, BTEC Higher level, professional qualifications (for example, teaching, nursing, accountancy)] | 1.76 | 1.46 – 2.11 | **<0.001** |
| Education Status [No qualifications] | 0.92 | 0.67 – 1.28 | 0.634 |
| Education Status [Other  vocational or work-related qualifications, other qualifications achieved in England or Wales, qualifications achieved outside England or Wales (equivalent not stated or unknown)] | 1.77 | 1.08 – 2.91 | **0.025** |
| Household Income [Between £25K and £39K] | 0.87 | 0.75 – 1.00 | 0.056 |
| Household Income [Less than £25K] | 0.81 | 0.69 – 0.96 | **0.012** |
| Household Income [Prefer not to say] | 1.11 | 0.83 – 1.49 | 0.481 |
| Device [non-mobile] | 1.09 | 0.95 – 1.24 | 0.234 |
| Age [35-49] | 1.13 | 0.96 – 1.34 | 0.144 |
| Age [50-64] | 1.19 | 0.99 – 1.42 | 0.057 |
| Age [65+] | 1.16 | 0.95 – 1.41 | 0.137 |
| Cafeteria Usage [3-4 days a week] | 0.94 | 0.49 – 1.80 | 0.848 |
| Cafeteria Usage [5-6 days a week] | 0.52 | 0.15 – 1.75 | 0.289 |
| Cafeteria Usage [Less than once a week] | 1.00 | 0.65 – 1.54 | 0.994 |
| Cafeteria Usage [Never] | 1.08 | 0.70 – 1.66 | 0.720 |
| Ecolabel Total Range [0] x Cafeteria Usage [3-4 days a week] | 1.34 | 0.54 – 3.28 | 0.527 |
| Ecolabel Total Range [1] x Cafeteria Usage [3-4 days a week] | 0.84 | 0.38 – 1.84 | 0.657 |
| Ecolabel Total Range [2] x Cafeteria Usage [3-4 days a week] | 0.84 | 0.40 – 1.76 | 0.643 |
| Ecolabel Total Range [3] x Cafeteria Usage [3-4 days a week] | 1.07 | 0.49 – 2.35 | 0.867 |
| Ecolabel Total Range [0] x Cafeteria Usage [5-6 days a week] | 0.32 | 0.03 – 3.61 | 0.357 |
| Ecolabel Total Range [1] x Cafeteria Usage [5-6 days a week] | 1.57 | 0.36 – 6.86 | 0.549 |
| Ecolabel Total Range [2] x Cafeteria Usage [5-6 days a week] | 1.40 | 0.34 – 5.77 | 0.639 |
| Ecolabel Total Range [3] x Cafeteria Usage [5-6 days a week] | 1.86 | 0.44 – 7.84 | 0.398 |
| Ecolabel Total Range [0] x Cafeteria Usage [Less than once a week] | 0.57 | 0.31 – 1.06 | 0.076 |
| Ecolabel Total Range [1] x Cafeteria Usage [Less than once a week] | 0.88 | 0.52 – 1.47 | 0.618 |
| Ecolabel Total Range [2] x Cafeteria Usage [Less than once a week] | 0.86 | 0.53 – 1.40 | 0.536 |
| Ecolabel Total Range [3] x Cafeteria Usage [Less than once a week] | 0.99 | 0.59 – 1.66 | 0.977 |
| Ecolabel Total Range [0] x Cafeteria Usage [Never] | 0.85 | 0.46 – 1.55 | 0.597 |
| Ecolabel Total Range [1] x Cafeteria Usage [Never] | 1.06 | 0.63 – 1.77 | 0.823 |
| Ecolabel Total Range [2] x Cafeteria Usage [Never] | 0.80 | 0.49 – 1.29 | 0.361 |
| Ecolabel Total Range [3] x Cafeteria Usage [Never] | 0.88 | 0.53 – 1.47 | 0.621 |
| **Random Effects** | | | |
| σ^2^ | 3.29 | | |
| τ_00_ _ResponseId_ | 0.65 | | |
| ICC | 0.17 | | |
| N _ResponseId_ | 2270 | | |
| Observations | 11350 | | |
| Marginal R^2^ / Conditional R^2^ | 0.042 / 0.200 | | |
